# Metabolic engineering to improve production of 3-hydroxypropionic acid from corn-stover hydrolysate in *Aspergillus* species

**DOI:** 10.1186/s13068-023-02288-1

**Published:** 2023-03-29

**Authors:** Ziyu Dai, Kyle R. Pomraning, Shuang Deng, Joonhoon Kim, Kristen B. Campbell, Ana L. Robles, Beth A. Hofstad, Nathalie Munoz, Yuqian Gao, Teresa Lemmon, Marie S. Swita, Jeremy D. Zucker, Young-Mo Kim, Kristin E. Burnum-Johnson, Jon K. Magnuson

**Affiliations:** 1 DOE Agile Biofoundry, Emeryville, CA 94608 USA; 2 Energy and Environment Directorate, Pacific Northwest National Laboratory, Richland, WA 99352 USA; 3 Earth and Biological Sciences Directorate, Pacific Northwest National Laboratory, Richland, WA 99352 USA

**Keywords:** Beta-alanine pathway, 3-hydroxypropionic acid, *Aspergillus niger*, *Aspergillus pseudoterreus*, Metabolic engineering

## Abstract

**Background:**

Fuels and chemicals derived from non-fossil sources are needed to lessen human impacts on the environment while providing a healthy and growing economy. 3-hydroxypropionic acid (3-HP) is an important chemical building block that can be used for many products. Biosynthesis of 3-HP is possible; however, low production is typically observed in those natural systems. Biosynthetic pathways have been designed to produce 3-HP from a variety of feedstocks in different microorganisms.

**Results:**

In this study, the 3-HP β-alanine pathway consisting of aspartate decarboxylase, β-alanine-pyruvate aminotransferase, and 3-hydroxypropionate dehydrogenase from selected microorganisms were codon optimized for *Aspergillus* species and placed under the control of constitutive promoters. The pathway was introduced into *Aspergillus pseudoterreus* and subsequently into *Aspergillus niger*, and 3-HP production was assessed in both hosts. *A. niger* produced higher initial 3-HP yields and fewer co-product contaminants and was selected as a suitable host for further engineering. Proteomic and metabolomic analysis of both *Aspergillus* species during 3-HP production identified genetic targets for improvement of flux toward 3-HP including pyruvate carboxylase, aspartate aminotransferase, malonate semialdehyde dehydrogenase, succinate semialdehyde dehydrogenase, oxaloacetate hydrolase, and a 3-HP transporter. Overexpression of pyruvate carboxylase improved yield in shake-flasks from 0.09 to 0.12 C-mol 3-HP C-mol^−1^ glucose in the base strain expressing 12 copies of the β-alanine pathway. Deletion or overexpression of individual target genes in the pyruvate carboxylase overexpression strain improved yield to 0.22 C-mol 3-HP C-mol^−1^ glucose after deletion of the major malonate semialdehyde dehydrogenase. Further incorporation of additional β-alanine pathway genes and optimization of culture conditions (sugars, temperature, nitrogen, phosphate, trace elements) for 3-HP production from deacetylated and mechanically refined corn stover hydrolysate improved yield to 0.48 C-mol 3-HP C-mol^−1^ sugars and resulted in a final titer of 36.0 g/L 3-HP.

**Conclusions:**

The results of this study establish *A. niger* as a host for 3-HP production from a lignocellulosic feedstock in acidic conditions and demonstrates that 3-HP titer and yield can be improved by a broad metabolic engineering strategy involving identification and modification of genes participated in the synthesis of 3-HP and its precursors, degradation of intermediates, and transport of 3-HP across the plasma membrane.

**Supplementary Information:**

The online version contains supplementary material available at 10.1186/s13068-023-02288-1.

## Background

The fuel and chemical products obtained from petroleum refineries have been essential in our daily life for more than a century. However, petroleum products are non-renewable and their production and use has contributed to widespread anthropogenic impacts on the earth’s atmosphere, lands, and oceans [1]. This has prompted the investigation of alternative routes to produce fuels and chemicals at low carbon intensity from renewable feedstocks [2]. Cost-effective utilization of existing industries to support the transition to alternative production routes for chemicals currently derived from petrochemical feedstocks will require synergistic efforts to maximize the output of biological systems and establish bioprocesses that are economically viable. To accomplish this, an Agile BioFoundry has been established to efficiently address the challenges associated with bioprocess development and engineer microorganisms for production of fuels and chemicals from renewable biomass feedstocks [3].

3-hydroxypropionic acid (3-HP) is a potential bioderived platform chemicals that can be converted into various commercial use chemicals, such as acrylic acid, malonic acid, 1,3-propanediol, and acrylamide, as well as direct use for production of biodegradable polymers [4, 5]. Chemical synthesis routes have been explored for 3-HP production, but high costs and adverse environmental impacts have limited chemical synthesis of 3-HP as a bulk chemical [6]. However, biological fermentation is a potential route for 3-HP production from renewable feedstocks and has been actively investigated for more than a decade [7, 8]. Naturally, there exist several 3-HP production processes, such as CO_2_ assimilation in *Chloroflexus aurantiacus* [9], cyanobacterium *Synechocystis* sp. [10, 11], glycerol oxidation in *Lactobacillus* sp. [12, 13], acrylic acid degradation in *Byssochlamys* sp. [14] or *Rhodococcus erythropolis* [15], and uracil catabolism in *Saccharomyces kluyveri* [16] or *E. coli* K-12 [17]. However, the efficiency of 3-HP production in native microorganisms is very low. Therefore, genetic engineering of non-native hosts with novel synthetic 3-HP production pathways is actively being evaluated to improve 3-HP production.

Glycerol oxidation through a coenzyme A-independent pathway was initially detailed by Bieble et al. for 1,3-propanediol production in *Clostridia* and *Enterobacteriaceae* and the NAD^+^-dependent aldehyde dehydrogenase that can convert 1,3-propanediol to 3-HP in *Klebsiella pneumoniae* by Raj et al. [18, 19]. *E. coli* or *K. pneumoniae* was genetically engineered and optimized for 3-HP production by combination of glycerol reduction and 3-HP production, which led to 70 to 80 g/l 3-HP production titer in fed-batch fermentations with glycerol as a carbon source [20, 21].

Biosynthetic routes to 3-HP via malonyl-CoA and β-alanine have been demonstrated in *E. coli* [22, 23] and further explored or optimized in various microorganisms such as *E. coli*, cyanobacteria, and *Saccharomyces cerevisiae*. For example, the malonyl-CoA pathway was introduced into *E. coli* for conversion of glucose to 3-HP, which resulted in 10 g/l 3-HP production in 36 h [24]. Similar results were observed for the same pathway after integration into the chromosomes of *S. cerevisiae* or *Schizosaccharomyces pombe* [25, 26]. The β-alanine 3-HP pathway consisting of aspartate decarboxylase, β-alanine pyruvate transaminase, and 3-HP dehydrogenase was constructed and examined in the *S. cerevisiae* with production titer of 13.4 g/l in controlled fed-batch fermentation [27]. Recently, we demonstrated that the β-alanine 3-HP pathway was functional in the acidophilic filamentous fungus *Aspergillus pseudoterreus* [28].

Filamentous fungi such as *Aspergillus* species are used industrially for organic acid production because of their ability to grow at very low pH (< 2.0) and produce secreted metabolites in nutrient-limited growth conditions, which eliminates the requirement of medium pH neutralization. *A. pseudoterreus* can secrete more than 80 g/l of itaconic acid in culture medium [29, 30] and *Aspergillus niger* can grow in more than 20% glucose or sucrose and convert more than 90% of the feedstock to citric acid [31, 32]. In this study, we explored and improved 3-HP production via the β-alanine pathway in the industrial *Aspergillus* species.

## Results

### Evaluation of 3-HP production in *Aspergillus pseudoterreus*

**Fig. 1:**
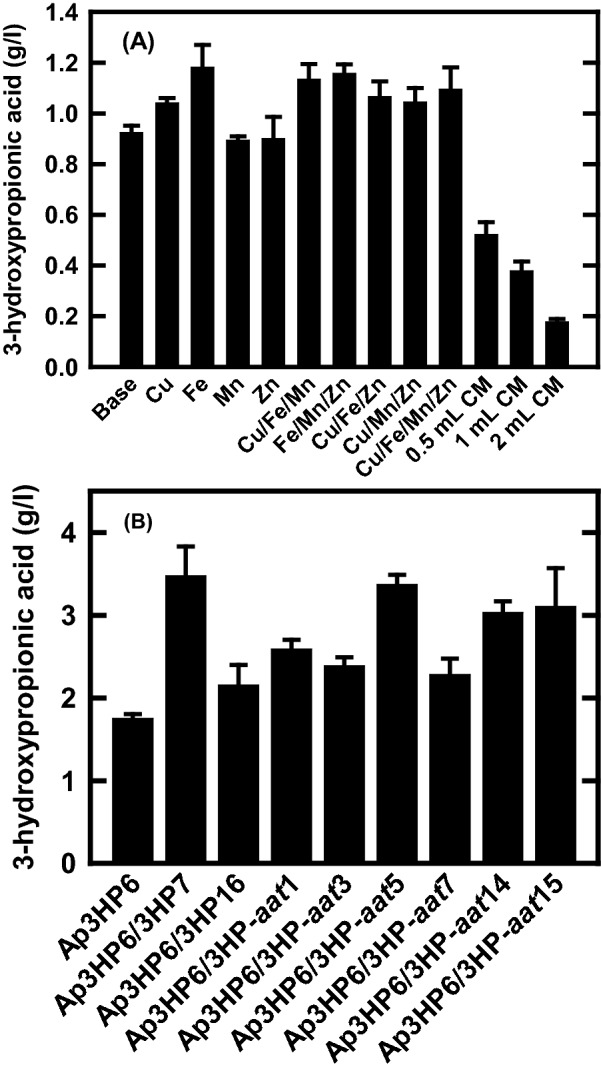
**A** The effects of different trace elements and CM on 3-HP production in the *A. pseudoterreus* transgenic strain Ap3HP6. The strain was inoculated in the mRDM medium with various amounts of components and grown at 30 °C and 200 rpm. Each data point is the average of three biological replicates. **B**. The effects of additional copies of 3HP pathway (3HP::*hph*: 3HP4070) or 3HP pathway + *aat1* overexpression (3HP::*hph/aat1*: 3HP4071) on 3-HP production in the *A. pseudoterreus* transgenic strain Ap3HP6 background. The strain was inoculated in the mRDM medium and grown at 30 °C and 200 rpm for 7 days. Each data point is the average of three biological replicates

A synthetic β-alanine pathway (3HP) for 3-HP production consisting of *Tribolium castaneum* aspartate decarboxylase (PAND), *Bacillus cereus* β-alanine-pyruvate aminotransferase (BAPAT), and *Escherichia coli* 3-hydroxypropionate dehydrogenase (HPDH) has been established and demonstrated in *S. cerevisiae* [27] and *A. pseudoterreus* [28]. In this study, we examined the effect of supplementation with trace elements (TE) and complex nutrients on 3-HP production in transgenic strain *A. pseudoterreus* Ap3HP6 that contains two copies of the β-alanine pathway (Additional file 1: Figs. S1 and S2) since the production medium B (RDM) was originally optimized for itaconic acid production [33]. Individual or combinations of TE were added to the base culture medium at up to 20-fold the original concentration. Figure 1A shows that both Cu and Fe enhance 3-HP production in modified production medium B (mRDM). However, no synergetic effects were observed when combinations of Cu, Fe, Mn, and Zn were added. In addition, supplementation with small amounts of nutrient-rich medium [0.5, 1, or 2 ml CM (complete medium)] substantially reduced 3-HP production.

We tested whether expression of the β-alanine pathway for 3-HP production was limiting by overexpressing additional copies of the pathway. A transgene expression cassette containing one (3HP) or one with the *aat* overexpression (3HP-*aat*) of the β-alanine pathway (Additional file 1: Figures S4 and S5) was randomly integrated into the chromosome of transgenic strain Ap3HP6. 3-HP titer in selected transgenic strains was increased up to 3.4 g/l, about twice the concentration of the parent strain (Fig. 1B). In addition to 3-HP, significant amounts of other organic acids such as aconitic acid and citric acid were produced by *A. pseudoterreus* [28], suggesting that it may not be an ideal acidophilic filamentous fungus for 3-HP production. Therefore, *A. niger*, an industrial species used for citric acid production, was examined as a host for 3-HP production.

### Evaluation of 3-HP production in *A. niger*

**Fig. 2:**
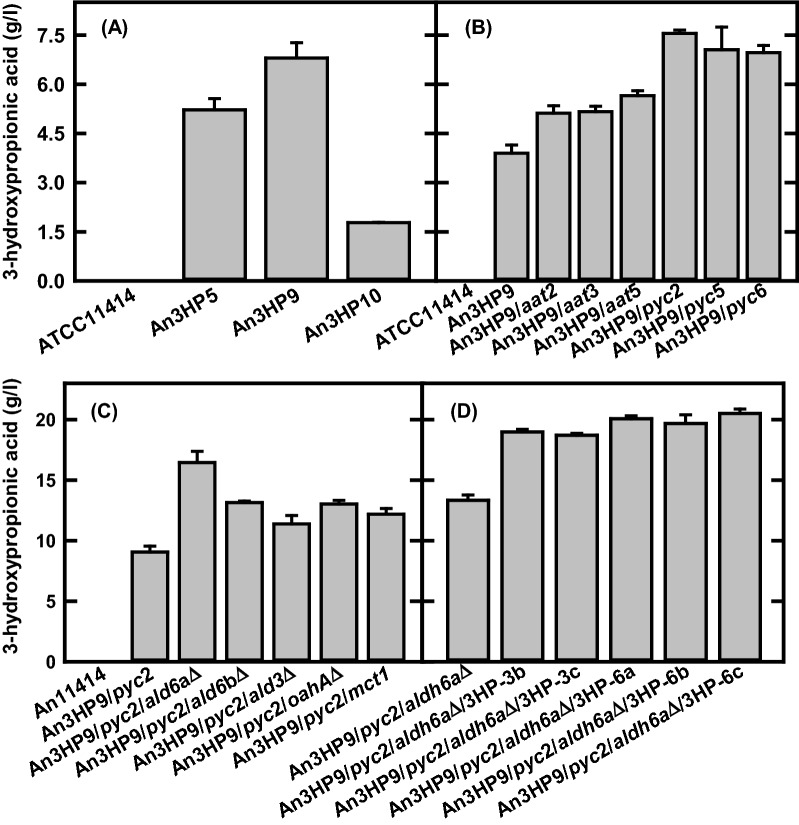
**A** 3-hydroxypropionic acid production (**A**) in the selected *A. niger* transgenic strains of An3HP5, An3HP9, and An3HP10; **B** in the selected *A. niger* transgenic strains with overexpression of pyruvate carboxylase (*pyc*) or cytosolic aspartate aminotransferase (*aat*) in An3HP9 strain; **C** in the *A. niger* transgenic strains with gene disruption of *ald6a*, *ald6b*, *ald3*, *oahA*, and *uga2* or overexpression of *mct1* gene in the *A. niger* transgenic strain An3HP9/*pyc2* strain; **D** in the selected *A. niger* transgenic strains with additional copies of β-alanine 3HP pathway. The strains were grown in the mRDM medium at 30 °C and 200 rpm for 7 days. The data are the average of three biological replicates

The same linearized transgene expression cassette (Additional file 1: Figure S1A) used in *A. pseudoterreus* [28] was randomly integrated into the chromosomes of *A. niger*. Three transgenic *A. niger* strains, An3HP5, An3HP9, and An3HP10, were selected for evaluation of 3-HP production in mRDM medium. The results in Fig. 2A show that transgenic strain An3HP9 produced the highest 3-HP titer, reaching 6.8 g/l in mRDM, a 200% increase over the highest titers produced by *A. pseudoterreus*. Prior to further genetic engineering in the An3HP9 strain, the copy number was estimated by Southern blotting analysis. The results (are) shown in the Additional file 1: Figure S6 shows that strain An3HP9 contains more than one copy of the β-alanine pathway for 3-HP production. The actual copies of insertion in An3HP9 were estimated as twelve by short-read whole genomic DNA sequencing (Table 1).

**Table 1:** Copy number estimate of genes in selected strains from whole genome sequencing

Strain	Copy number estimate					
	Tc*panD*	Bc*bapat*	Ec*hpdh*	*aat1*	*pyc2*	*ald6a*
ATCC 11414	0	0	0	1	1	1
An3HP9/*aat1*	12	13	11	5	1	1
An3HP9/*pyc2*/*ald6a*∆/3HP-6	26	29	25	1	8	0

### Multi-omics analysis of 3-HP production in *Aspergillus species*

**Fig. 3:**
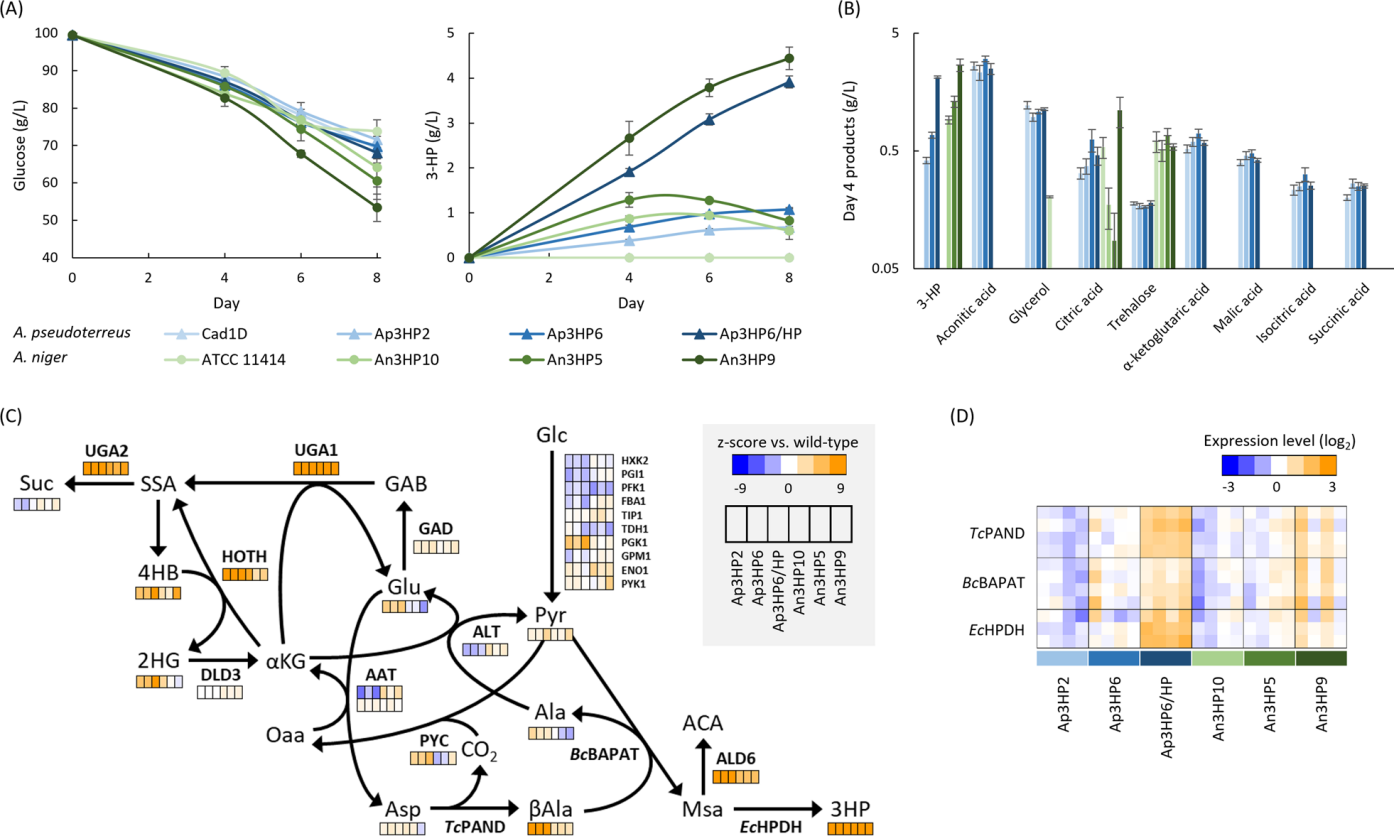
Multi-omic analyses of 3-HP production in *Aspergillus* species. **A**) Shake-flask cultivation of *Aspergillus* species engineered to produce 3-HP. **B** Extracellular metabolites detected and quantified by GC–MS at day four. **C** Protein and intracellular metabolite levels relative to the un-engineered parent strains at day four. **D** Targeted peptide quantification of the heterologous pathway proteins at day four. Multiple boxes for a single protein indicate different targeted peptides. The data represent four biological replicates

In both *A. pseudoterreus* and *A. niger*, transformants of the same β-alanine pathway for 3-HP production were isolated that produce a broad range of 3-HP titers. We compared transformants of both species during time-course cultivation in shake flasks in mRDM (Fig. 3A). In some cases, 3-HP titer declined later in the cultivation consistent with previous observations of 3-HP degradation catalyzed by the methylmalonate semialdehyde dehydrogenase *ald6* [28]. We therefore collected biomass and supernatant samples at day four, prior to decrease in titer, to assess the impacts of 3-HP production on metabolism by global and targeted proteomics, and intra- and extracellular metabolomics. While the range of 3-HP yields is comparable for the transformants obtained from the two *Aspergillus* species, the spectrum of co-products is dissimilar. Aside from 3-HP, from the panel of metabolites quantified, only trehalose and citric acid were detected in the *A. niger* fermentation broth. In contrast, *A. pseudoterreus* produced a wide variety of contaminating co-products that include glycerol and most of the tricarboxylic acid (TCA)-cycle derived organic acids (Fig. 3B). Targeted peptides designed for the heterologous enzymes in the β-alanine pathway were used to compare expression level between the species (Fig. 3C) and confirmed that the pathway is expressed at a higher level in strains that produce more 3-HP.

Global proteomics and metabolomics were used to assess the impact of increasing 3-HP production on metabolism. Metabolomics revealed that in both species intracellular β-alanine, 3-HP, and 4-hydroxybutyrate were significantly increased in all transformed strains. Proteomic analysis identified ALD6 as significantly upregulated in all engineered strains indicating degradation via the pathway intermediate malonate semialdehyde is a conserved aspect of 3-HP metabolism in *Aspergillus* species. Proteins involved in the GABA shunt (UGA1 and UGA2) and a hydroxyacid-oxoacid transhydrogenase involved in conversion of α-ketoglutarate to succinate semialdehyde and mobilization of 4-hydroxybutyrate [34] are also responsive to 3-HP production and upregulated in all engineered strains (Fig. 3D).

Most enzymes along the ideal path from glucose to 3-HP do not exhibit a consistent response to the presence of the heterologous 3-HP production pathway. However, in the highest producing *A. niger* strains alanine, glutamate, and aspartate are depleted, suggesting that nitrogen pools may be stressed in this host, while in *A. pseudoterreus* glutamate and alanine are accumulated and both alanine transaminase and aspartate aminotransferase are downregulated.

### Augmenting metabolic flux to precursor molecules required for 3-HP production in *A. niger*

When directly compared, *A. niger* produced 3-HP with fewer co-product contaminants than *A. pseudoterreus*. We therefore focused on the improvement of 3-HP yield from glucose in *A. niger*. Carbon efficient flux toward 3-HP relies on the precursor metabolites, oxaloacetate and aspartate. Omics analysis indicated that nitrogen pools that support flux through these metabolites may be strained and that the expression of pyruvate carboxylase, a critical step for carbon efficiency, may be limited in some strains. Flux toward oxaloacetate and aspartate was examined for their contribution to 3-HP production in *A. niger* by overexpression of pyruvate carboxylase (*pyc*) and aspartate aminotransferase (*aat*). cDNA of *aat* or *pyc* under the control of *tef1* (*tef1*p-*aat*, Additional file 1: Figure S7A), or *mbfA* promoter (*mbfA*p-*pyc*, Additional file 1: Figure S7B) or the combination of *aat* and *pyc* (*tef1*p-*aat*-*pgk*t-*pyc*-*mbfA*p, Additional file 1: Figure S7C) was integrated into transgenic strain An3HP9. Transformants were evaluated for 3-HP production (Additional file 1: Figure S8) and strains with improved titer were purified (Fig. 2B). Overexpression of *aat* increased titer of 3-HP up to 43%, while *pyc* overexpression increased titer by as much 93% compared to the original transgenic An3HP9 strain. This indicates that flux toward beach-head metabolites for 3-HP is limiting in An3HP9; however, no synergistic effects were observed in transgenic strains overexpressing both *aat* and *pyc* together (Additional file 1: Figure S8C). The copy number of *aat* or *pyc* in transgenic strains An3HP9/*aat*5 and An3HP9/*pyc*2 (Table 1) was estimated as five and eight by short-read whole genomic DNA sequencing, respectively.

### Effects of genes involved in metabolism of β-alanine pathway intermediates and 3-HP transport in ***A. niger***

Recently, we identified genes potentially involved in metabolism of 3HP pathway intermediates via multi-omics studies in *A. pseudoterreus* [28] and confirmed many of these as targets in *A. niger* (Fig. 3). In this study, the *A. niger* homologs of malonate semialdehyde dehydrogenase (jgi|Asppseute1|414254 [Ap*ald6*] and jgi|Asppseute1|497789 [Ap*ald3*]), succinate semialdehyde dehydrogenase (jgi|Asppseute1|447301 [Ap*uga2*]), a putative 3-HP transporter identified from *A. pseudoterreus* (jgi|Asppseute1|474223[Ap*mct1*]), and *A. niger* oxaloacetate hydrolase (An*oahA*) were examined for their effect on 3-HP production in the transgenic An3HP9/*pyc*2 strain (Additional file 1: Figure S7E and S9). Single gene homologs were identified for all the targets except Ap*ald6*, where two homologs were identified in *A. niger* (An*ald6a* and An*ald6b*). The results in Fig. 2C show that disruption of An*ald6a* in strain 3HP/*pyc*2 increased 3-HP titer by 83% to 16.5 g/l, while disruption of An*ald6b* and An*ald3* increased titer by 45% and 26%, respectively suggesting that, while An*ald6a* is likely the major malonate semialdehyde dehydrogenase, all three contribute to directing flux away from 3-HP. In contrast, when An*uga2* was deleted, 3-HP production decreased by 37%. In this strain, growth and sugar conversion rate was decreased and the specific yield of 3-HP increased. This suggests that yield of 3-HP from sugars may be improved by limiting flux through the GABA shunt, but that deletion of the pathway entirely is overly detrimental to growth. Disruption of oxaloacetate hydrolase (An*oahA*) increased the titer of 3-HP by 45% without impacting growth suggesting that flux toward oxalic acid, a product secreted by *A. niger* [35], represents a substantial loss in yield of 3-HP. A monocarboxylate transporter (An*mct1,* Additional file 1: Figure S7E), where the homolog in *A. pseudoterreus* responds to the presence of intracellular 3-HP [28], was overexpressed, resulting in a 35% improvement in 3-HP suggesting that transport across the plasma membrane may limit 3-HP production.

### Effects of additional copies of β-alanine pathway on 3-HP production in ***A. niger***

Our initial set of *A. niger* and *A. pseudoterreus* strains expressing various levels of the 3-HP production pathway demonstrated that a higher expression level of the β-alanine pathway genes increased the yield of 3-HP (Fig. 3). To determine whether flux through the β-alanine pathway was still a limiting factor for the yield, we further increased expression by randomly integrating a new transgene expression cassette (Additional file 1: Figure S10E) into the highest producing An3HP9/*pyc*2/*ald6a*Δ strain. Transformants with higher 3-HP titer than the parent were identified and the best performing single-spore isolate produced 20.5 g/l 3-HP, a 53% improvement over the parent strain (Fig. 2D).

Short-read whole genome sequencing was performed on selected strains at critical points in the construction lineage to estimate copy number of the randomly integrated plasmids (Table 1). We found that approximately 12 copies of the β-alanine pathway were randomly integrated into the *A. niger* chromosomes during construction of strain An3HP9 and that seven additional copies of *pyc2* were integrated into the genome in strain An3HP9/*pyc2*. In the highest producing strain (An3HP9/*pyc2*/*ald6a*∆/3HP-6), we confirmed deletion of *ald6a* and found that an additional 15 copies of the β-alanine pathway were inserted into the genome bringing the total copy-number of the β-alanine pathway to approximately 27 copies.

### Optimization of culture conditions for 3HP production

Culture conditions for citric acid by *A. niger* have been optimized and are dependent on pH, as well as carbon, nitrogen, and manganese concentrations [31]. In this study, the effects of pH, manganese, and nitrogen on 3-HP production were examined with initially genetically engineered strain An3HP9 or 3HP9/*pyc*2. The effects of citric acid production (CAP) versus mRDM medium were examined in the first *A. niger* transgenic strain An3HP9 by growing them in either CAP medium or mRDM with the pH ranging from 2.0 to 3.4 since pH 2.0 is the optimal pH for CAP. 3-HP titer in CAP medium was 2.7 g/L, significantly lower than in mRDM across the pH spectrum. (Additional file 1: Figure S1A). In mRDM, the spore germination rate increased with increasing pH up to 30.7% at pH 3.4 (Additional file 1: Figure S1B) and coincided with increased consumption of glucose resulting in an insignificantly lower yield of 3-HP.

**Fig. 4:**
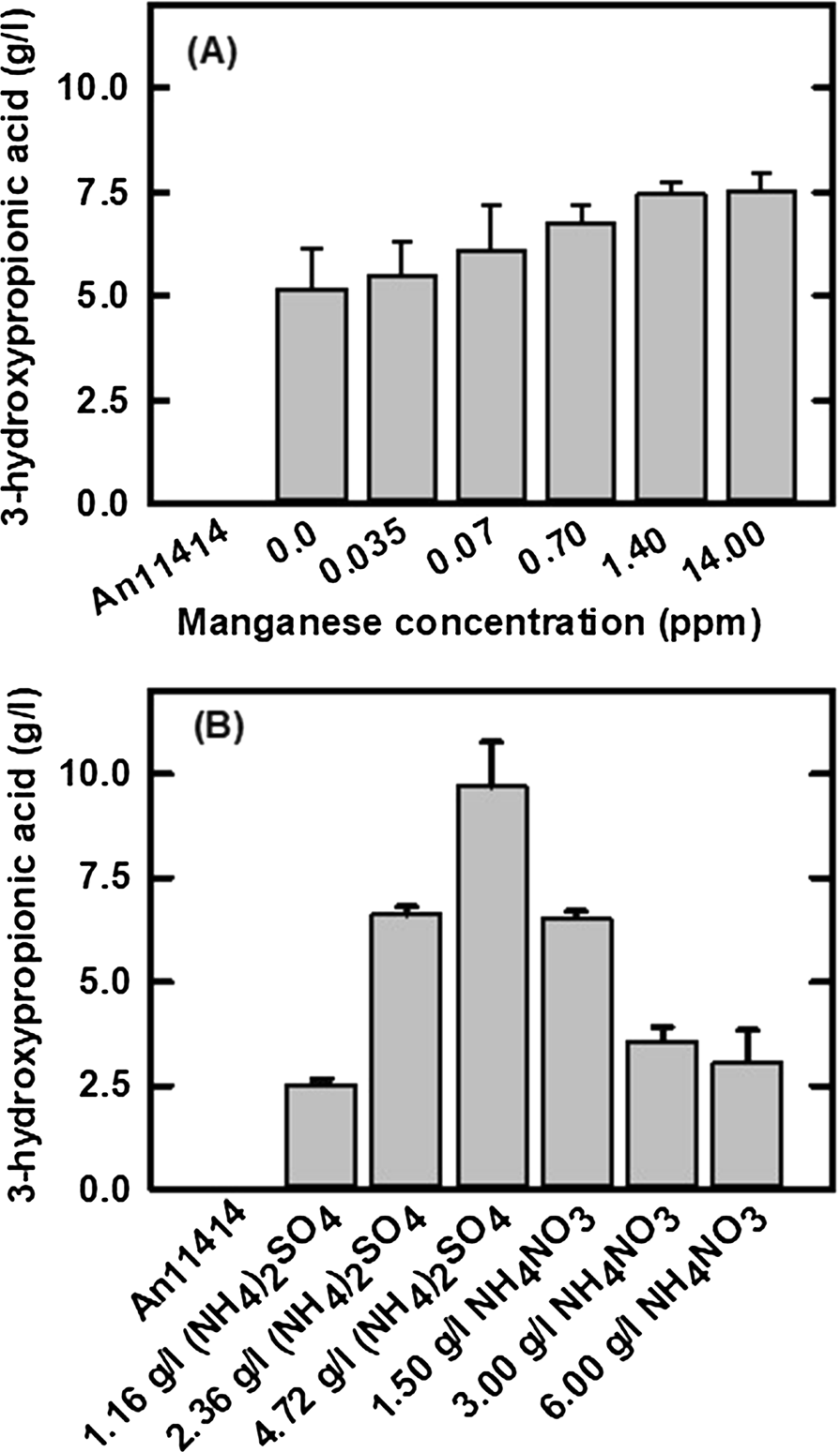
The effects of manganese and nitrogen sources on 3-hydroxypropionic acid production in *A. niger* transgenic strain An3HP/*pyc*2 grown at 30 °C and 200 rpm for 7 days. **A** The effects of manganese on 3-HP production and **B** the effects of different nitrogen sources on 3-HP production. The data are the average of three biological replicates

To achieve maximal 3-HP production, the effects of manganese on 3-HP production were evaluated in the transgenic strain An3HP9/*pyc*2 strain, which contains 8 copies of *pyc* transgene overexpression in An3HP9 with 90% improvement in 3-HP production. When the strain was grown with 0 ~ 0.035 ppm manganese, 3-HP titer reached 5.5 g/l but when manganese was increased to 0.07, 0.7, 1.4, 14.0 ppm manganese, the 3-HP titer increased to 6.1, 6.7, 7.4, and 7.5 g/l, respectively, suggesting that the required manganese level to support 3-HP metabolism is around 1.4 ppm, about 140-fold higher than that required for citric acid production in *A. niger* (Fig. 4A). At all manganese concentrations, the yield of 3-HP was not significantly different while the yield of biomass trended downward with increasing manganese suggesting that the increased titer is due to differences in the rate of 3-HP production. The effect of nitrogen concentration and source, in the form of (NH_4_)_2_SO_4_ or NH_4_NO_3_, on 3-HP production in the An3HP9/*pyc*2 strain was also examined (Fig. 4B). 3-HP production increased from 2.5 to 9.7 g/l when the strain was grown in mRDM with 1.16, 2.36, or 4.72 g/l (NH_4_)_2_SO_4_, respectively. Increasing nitrogen increased the sugar conversion rate, but overall yield was still significantly higher at the greatest (NH_4_)_2_SO_4_ concentration (*p* < 0.01). The β-alanine pathway to produce 3-HP is dependent on balanced flux through three transaminases [AAT, alanine transaminase (ALT), and BAPAT] and excess nitrogen may be required to support efficient flux through these reactions. When the strain was grown in mRDM with increasing concentrations of NH_4_NO_3_, 3-HP production decreased suggesting that the impact of NO_3_ as a nitrogen source may be detrimental to 3-HP production. The optimal nitrogen source for batch 3-HP production in shake-flasks was 4.72 g/l of (NH_4_)_2_SO_4_, which corresponds to a C/N ratio of 40.

### Production of 3-HP from lignocellulosic feedstock derived sugars

**Fig. 5:**
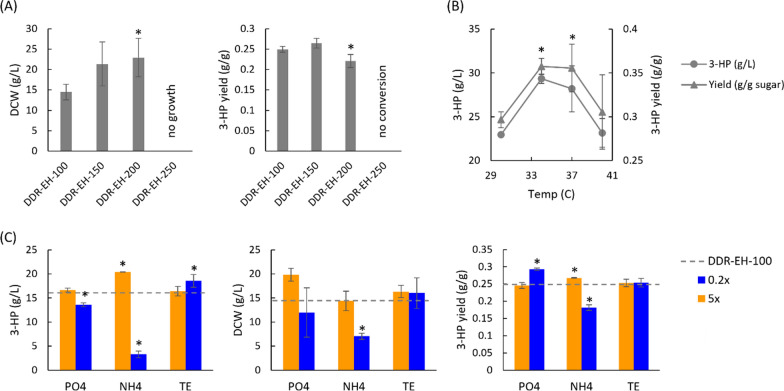
Optimization of 3-HP production in RDM with sugars from DDR-EH using *A. niger* strain An3HP9/*pyc*-2/*ald6a*Δ at 200 rpm for 7 days. **A** Ability of *A. niger* to germinate and grow in increasing DDR-EH concentrations from 100 to 250 g/L total sugar. **B** 3-HP production in DDR-EH-100 at 30, 34, 37, and 40 °C. **C** 3-HP production in DDR-EH-100 with limiting (0.2x) and excess (5x) concentrations of phosphate, ammonia, and trace elements. The data are the average of three biological replicates and is corrected for evaporative loss. Asterisks indicate statistically significant differences (*p* < 0.05) from the baseline condition (RDM with 100 g/L total sugars from DDR-EH at 30 °C)

In addition to purified glucose as a carbon source for 3-HP production, we considered sugars derived from lignocellulosic biomass as a feedstock. Corn stover was subjected to dilute alkali deacetylation prior to disk refining and enzymatic hydrolysis (DDR-EH) to release glucose and xylose monomers [36, 37]. We investigated conversion of sugars produced by the DDR-EH process using both An3HP9/*pyc*2/*ald6a*Δ and An3HP9/*pyc*2/*ald6a*Δ*/*3HP-6 strains with the highest 3-HP production titers. We initially tested 3-HP production of An3HP9/*pyc*-2/*ald6a*Δ strain in RDM to provide essential micro and macro-nutrients with increasing concentrations of sugars from the DDR-EH process to identify limits on conversion due to toxicity. We found that *A. niger* was able to germinate and grow in up to 200 g/L total sugars from the DDR-EH process (Fig. 5A) with a significantly lower yield of 3-HP at the highest sugar concentration where growth was maximized. We next looked at the impact of temperature on conversion in DDR-EH and found that 34–37 °C significantly improved both 3-HP yield and titer with a maximum of 29.4 g/L 3-HP and 0.35 C-mol 3-HP C-mol^−1^ sugars (Fig. 5B). Sugars produced using the DDR-EH process contain a wide variety of characterized and unknown metabolites. To determine whether phosphate, nitrogen, and trace elements (TE) required for growth and production are present at necessary levels in DDR-EH, we modified their concentration up and down by fivefold to represent limiting and excess concentrations (Fig. 5C). We found that reducing phosphate or nitrogen concentration reduced biomass production and 3-HP titer suggesting that DDR-EH needs to be supplemented with both of these macronutrients to maximize 3-HP production. However, reduction of TE significantly improved 3-HP titer (*p* < 0.05) while excess TE had no impact on growth or productivity suggesting that TE in DDR-EH may be present at necessary concentrations without supplementation. When excess nitrogen was supplied, 3-HP titer and yield were both significantly improved without increasing biomass suggesting that supply of precursors for the multiple transaminases of the β-alanine pathway may not be optimized and flux is aided by the presence of excess nitrogen.

**Fig. 6:**
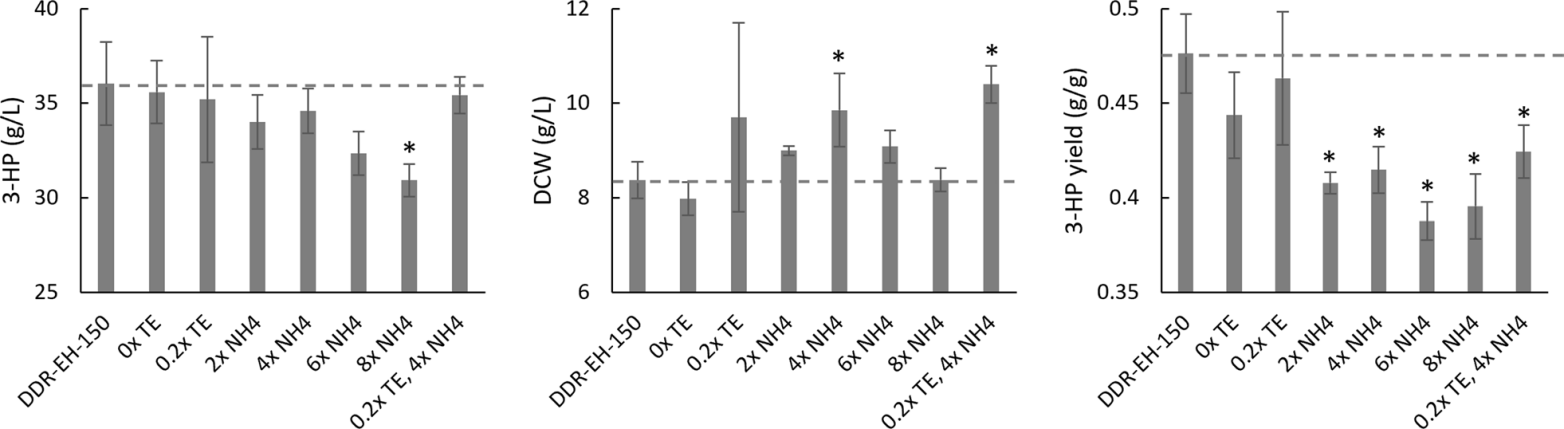
The impact of nitrogen and trace elements on 3-HP production in RDM with 150 g/L sugars from DDR-EH using *A. niger* strain An3HP9/*pyc*-2/*ald6a*Δ/3HP-6 at 200 rpm and 34 °C for 7 days. The data are the average of three biological replicates and are corrected for evaporative loss. Asterisks indicate statistically significant differences (*p* < 0.05) from the baseline condition (RDM with 150 g/L total sugars from DDR-EH at 34 °C)

Some of the potential improvements in cultivation conditions to increase yield, rate, or cost for production of 3-HP from DDR-EH derived sugars were combined and tested with the highest yield An3HP9/*pyc2*/*ald6a*∆/3HP-6 strain. The standard concentration of sugars was increased to 150 g/L (97.5 g/L glucose and 52.5 g/L xylose) and the temperature increased to 34 °C prior to retesting the impacts of nitrogen and trace elements (Fig. 6). With the combined improvements in the standard RDM with 1 × TE and 1 × N [2.36 g/l (NH_4_)_2_SO_4_], 36.0 g/l 3-HP was produced, and the yield was improved to 0.48 C-mol 3-HP C-mol^−1^ sugars. We found that reducing or completely eliminating the addition of TE from the standard RDM did not have a significant impact on growth or 3-HP production. Increasing the concentration of nitrogen tended to increase the amount of biomass produced and, in all cases, significantly decreased the yield of 3-HP.

## Discussion

**Fig. 7:**
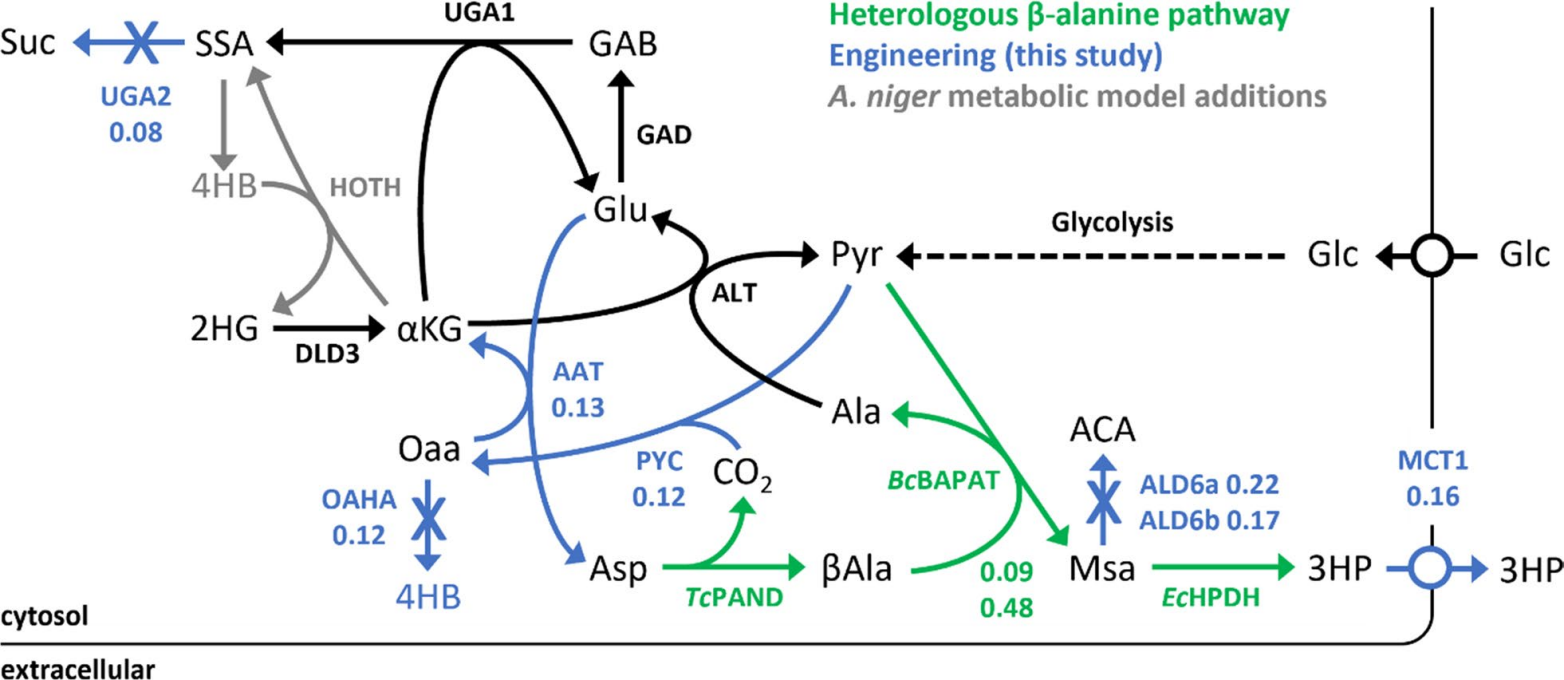
Summary of strain improvements for 3-HP production in *A. niger*. Yields achieved by overexpression or deletion (indicated by an ‘x’) of genes involved in 3-HP production. All yields achieved are in the 3HP-PYC background except for the β-alanine base and final pathway yields and the PYC yield

The biosynthesis of 3-HP as a secreted monomer or an intracellular polymer has been examined in a variety of microorganisms and from various feedstocks. Production of monomeric 3-HP as a precursor for commodity chemical production has been proposed and developed primarily using sugars or glycerol as feedstocks, both of which have the potential to offer economic and green-house gas emission benefits compared to petroleum derived feedstocks [38]. Production of 3-HP from glycerol as a feedstock using bacterial hosts has made substantial gains toward economic viability [39, 40]; however, high-yield production from sugar feedstocks has been more challenging [41] with only modest yields achieved in hosts that require expensive nutrient supplements and are limited to production of 3-hydroxypropionate near neutral pH production conditions rather than 3-hydroxypropionic acid (Table 2). We therefore engineered the β-alanine pathway, which was initially alluded to in patents granted to Cargill/Novozymes [42] and later established academically in the yeast *S. cerevisiae* [27], in the filamentous fungal hosts *A. pseudoterreus* and *A. niger*, which have been demonstrated at scale for industrial production of organic acids at acidic pH (< 2.0) and are capable of converting mixed sugar feedstocks with minimal nutrients and without pH neutralization requirement. After several DBTL (design-build-test-learn) cycles and culture optimizations, the 3-HP production in the best *A. niger* transgenic strain reached 36.0 g/l with the DDR-EH derived sugars from corn stover, which is summarized in Fig. 7.

**Table 2:** Nutritional requirements for production of 3-HP from sugar monomers

Host	Carbon source	Titer (g/L)	Rate (g/Lh)	Yield (g/g)	NH_4_ (g/L)	PO_4_ (g/L)	Additives	pH	Refs.
*E. coli*	Glucose (pure)	40.6	0.56	0.19	3.0	9.8	Vitamins, IPTG	7.0, NH_3_	[65]
*S. pombe*	Glucose (pure)	11.2	0.12	0.12	5.0	2.2	Vitamins	5.0, NH_3_	[50]
*S. cerevisiae*	Glucose (pure)	13.7	0.17	0.17	15.0	6.0	Vitamins	5.0, NaOH	[27]
*S. cerevisiae*	Glucose (pure)	9.2	0.13	0.09	15.0	6.0	Vitamins	3.5, NaOH	[27]
*S. cerevisiae*	Glucose (pure)	9.8	0.09	0.13	15.0	6.0	Vitamins	5.0, NaOH	[25]
*S. cerevisiae*	Xylose (pure)	7.4	0.06	0.29	15.0	3.0	Vitamins	5.0, NaOH	[51]
*C. glutamicum*	Glucose/xylose (pure)	54.8	0.76	0.49	24.0	2.5	Vitamins, IPTG, corn steep liquor	7.2, NH_3_	[52]
*E. coli*	Glucose/xylose (pure)	37.6	0.63	0.17	4.0	13.5	Vitamins, IPTG, yeast extract	6.8, NH_3_	[53]
*E. coli*	Glucose/xylose (pure)	53.7	0.63	0.13	4.0	13.5	Vitamins, IPTG, yeast extract	6.8, NH_3_	[54]
*E. coli*	Glucose/xylose (pure)	29.7	0.54	0.36	4.0	13.5	Vitamins, IPTG, yeast extract	6.8, NH_3_	[55]
*A. niger*	Glucose/xylose (hydrolysate)	36.0	0.21	0.48	2.4	0.1	–	<2.0, no control	This work

In the first design-build-test-learn (DBTL) cycle, the β-alanine pathway was functionally expressed in the filamentous fungus *A. pseudoterreus* and genes related to 3-HP degradation enzymes (Ap*ald6*, Ap*ald3*, Ap*uga2*) and monocarboxylate transport across the plasma membrane (Ap*mct1*) were identified via transcriptomic and proteomic analyses [28]. The lessons we learned from the first DBTL cycle in *A. pseudoterreus* facilitated the transfer of the β-alanine pathway into the *A. niger*, citric acid production strain [43]. The same transgene expression cassette used in *A. pseudoterreus* was randomly chromosomally integrated into *A. niger* and conferred a higher initial titer of 3-HP. Analysis of both species revealed that increased 3-HP yield correlates to copy number and expression level of the β-alanine pathway, which may explain the higher initial titers in the *A. niger* strains. Production of other organic acids, such as citric acid, was below the detection limit in *A. niger* allowing more carbon to be routed toward 3-HP production.

The effects of increasing the intracellular pool of precursor metabolites such as oxaloacetate and aspartic acid on 3-HP production were first evaluated by increasing the expression of *aat1*, or *pyc* in the An3HP9 transgenic strain with about 30 to 90% improvements in 3-HP titer in the selected transgenic strains. Elimination of carbon fluxes competing with β-alanine pathway metabolic intermediates oxaloacetate, malonic semialdehyde, and glutamate/α-ketoglutarate was also examined in transgenic strain An3HP9/*pyc*2. All exhibited positive effects on 3-HP production and 83% improvement in titer of 3-HP production were observed with the disruption of *ald6a* gene. Previously we observed significant improvements in flux toward 3-HP in *A. pseudoterreus* after disruption of Ap*ald6* [28] that are consistent with results from the homolog in *Candida albicans* [44]. This highlights that while *ald6a* may be the major contributor to the pathway competition, *A. niger* encodes at least three enzymes capable of metabolizing the 3-HP precursor malonic semialdehyde that may need to be simultaneously disrupted to maximize yield of 3-HP.

To test for the possibility of reactions limiting the final steps in conversion of pyruvate to 3-HP we increased the expression level of the heterologous enzymes in the β-alanine pathway in the An3HP9/*pyc*2/*ald6a*Δ strain by increasing the chromosomally integrated copy-number of the pathway from 12 to 27 (Fig. 2D, Table 1). We also overexpressed a putative 3-HP transporter identified from our previous work [28] to test whether export may be limiting (Fig. 2C). In both cases, increased expression improved the yield of 3-HP suggesting that the expression level of critical enzymes for 3-HP production is still limiting in the engineered strains and highlighting the need for novel metabolic engineering tools in *Aspergillus* species capable of increasing expression level of enzymes orders of magnitude beyond what is currently possible.

In addition to modification of pathway enzymes in the transgenic strains, culture conditions play an essential role in directing metabolic flux toward 3-HP. The initial pH in *A. niger* cultures for citric acid production is 2.0 and manganese is limited to 10 ppb [31]. Here, we found that an initial pH range from 3.4 to 2 did not significantly affect 3-HP yield in the An3HP9 strain though the spore germination frequency is higher at pH 3.4. 3-HP is produced in CAP medium which supports overflow metabolism, but to a lesser extent than in the mRDM medium. Therefore, the effects of the mRDM medium on 3-HP production were further optimized by alternation of individual components. Maintenance of manganese at a very low concentration (~ 10 ppb) is required to support high-yield production of citric acid in *A. niger* [31, 45] as well as itaconic acid in *A. pseudoterreus* [46]. In this study, however, we found that 100-fold higher concentrations of manganese in the culture medium support 3-HP production highlighting the difference in metabolism between production of 3-HP and the organic acids typically produced by these organisms. Tolerance, or even a requirement for higher concentrations of trace elements for 3-HP production, avoids some of typical challenges with organic acid production by *Aspergilli* whereby micro-nutrients leaching from metal fermentation vessels or present as contaminants in feedstock sugars negatively impact yield [45].

An essential component of economically viable 3-HP production is utilization of inexpensive feedstocks [4]. The 3-HP production on a DDR-EH derived sugars from corn stover was evaluated and optimized with the higher 3-HP production transgenic strains. We found that raw DDR-EH requires supplementation macro-nutrients (nitrogen and phosphate) to support growth and 3-HP production by *A. niger*, but that the trace elements added to RDM can be eliminated as an additive to reduce cost. The optimal temperature for 3-HP production was around 34 °C, which is consistent with optimal temperatures for enzyme activities of PAND (50 °C) of *T. castaneum* [47], BAPAT (35 °C) of *B. cereus* [48], and HPDH (37 °C) of *E. coli* K12-TG1 [49]. Increasing the concentration of (NH_4_)_2_SO_4_ to the optimal level in RDM supported more growth but reduced the yield of 3-HP in DDR-EH suggesting a lower concentration of nitrogen can be used to further reduce production costs.

Engineering efforts to produce 3-HP in fungi have demonstrated modest yields from pure glucose and xylose but typically supplement with vitamins [25, 27, 50, 51]. Efforts have been made to produce 3-HP in the yeast *S. cerevisiae* in acidic conditions below the pKa of 3-HP, however yield was nearly half that achieved at pH 5 [27]. Engineering efforts to produce 3-HP from mixed sugars in bacteria (*E. coli* and *C. glutamicum*) have also been successful but typically require growth at neutral pH and supplementation with vitamins and a complex nutrient source such as yeast extract or corn-steep liquor [52–55]. Production of 3-HP using *A. niger* alleviates many of the economic issues presented by model yeast and bacterial hosts by eliminating the need to supplement with costly vitamins and complex nutrients and allowing for production as a free acid amenable to low-cost purification strategies (Table 2).

## Conclusions

In summary, the β-alanine pathway functions and enables high-yield 3-HP production at acidic pH from low-cost sugars derived from corn stover in the industrial filamentous fungus *A. niger*. Results from a DBTL cycle comparing multiple species and strains in conjunction with optimization of cultivation conditions dramatically increased the yield of 3-HP to 48% of the no-growth theoretical yield from a corn-stover feedstock. This work establishes *Aspergillus* species as a platform for commercial production of renewable 3-HP as a precursor for a variety of fossil-derived chemicals including 1,3-propanediol, acrylic acid, methyl acrylate, acrylamide, and acrylonitrile. Future work focused on stacking of genetic improvements identified herein a single transgenic strain and scale-up will de-risk commercial production of renewable 3-HP and support the transition of commodity-scale chemical synthesis to lower green-house gas emitting processes.

## Methods

### Strains and media

The *Escherichia coli* strain Top10 was used for routine plasmid DNA preparation. *A. pseudoterreus* (ATCC 32359) and *A. niger* (ATCC 11414) from the American Type Culture Collection (Rockville, MD, USA) were grown on complete medium (CM) or potato dextrose agar (PDA) plates at 30 °C for culture maintenance and spore preparation. About 1 × 10^4^ to 1 × 10^5^ spores were inoculated on CM agar (petri dish) plates and incubated for four days at 30 °C. Spores were harvested by washing with 5–10 ml sterile 0.4% Tween 80 (polyoxyethylenesorbitan monooleate) and pelleted by centrifugation at 2500 g for 5 min. The spores were re-suspended in sterile 0.4% Tween 80 and enumerated with a hemocytometer. Aliquots of the resulting spore suspension (about 10^8^ ~ 10^9^ spores/ml) were used to inoculate different agar-plates or liquid cultures. The preparation of PDA, CM, and minimal medium (MM) followed the description of Bennett and Lasure [56]. All strains used in this study are shown in Table 3.

**Table 3:** Parent and transgenic strains of *A. pseudoterreus* and *A. niger* used in this study

Strain name	Relevant genotype	References
	**Parent strain:** *Aspergillus pseudoterreus* ATCC32359 (AP)	
AP*cad1*Δ	*cad1*::*hph*	[28, 59]
Ap3HP2	*cad*::**3HP**[*gpdA*p-*panD*-*elf3*t-*bapat*-*eno1*p, *gpdA*p-*hpdh*-*trpC*t, *ptrA*, (1 copy)*]	[28]
Ap3HP6	*cad*::**3HP**[*gpdA*p-*panD*-*elf3*t-*bapat*-*eno1*p, *gpdA*p-*hpdh*-*trpC*t, *ptrA*, (2 copies)]	[28]
Ap3HP6/HP7 or 16	*cad*::**3HP**[*gpdA*p-*panD*-*elf3*t-*bapat*-*eno1*p, *gpdA*p-*hpdh*-*trpC*t, *ptrA*, (2 copies)]/**3HP**[*gpdA*p-*panD*-*elf3*t-*bapat*-*eno1*p, *gpdA*p-*hpdh*-*trpC*t, *hph,* (unknown copies)]	This work
Ap3HP6/HP-*aat* series	*cad*::**3HP**[*gpdA*p-*panD*-*elf3*t-*bapat*-*eno1*p, *gpdA*p-*hpdh*-*trpC*t, *ptrA*, (2 copies)]//**3HP-*aat*** [*gpdA*p-*panD*-*elf3*t-*bapat*:*eno1*p, *gpdA*p-*hpdh*-*trpC*t, *hph*, *tef1*p:*aat*:*pgk1*t, (unknown copies)]	This work
	**Parent strain:** *Aspergillus niger* ATCC 11414 (An)	
ATCC 11414	wild-type (parent)	[31, 43]
An3HP5	**3HP**[*gpdA*p*-panD-elf3*t*-bapat-eno1*p*, gpdA*p*-hpdh-trpC*t*, ptrA,* (unknown copies)]	This work
An3HP9	**3HP**[*gpdA*p*-panD-elf3*t*-bapat-eno1*p*, gpdA*p*-hpdh-trpC*t*, ptrA*, (12 copies)**]	This work
An3HP10	**3HP**[*gpdA*p*-panD-elf3*t*-bapat-eno1*p*, gpdA*p*-hpdh-trpC*t*, ptrA* (unknown copies)]	This work
An3HP9/*aat2, 3, or 5*	**3HP**[*gpdA*p*-panD-elf3*t*-bapat-eno*p*, gpdA*p*-hpdh-trpC*t*, ptrA*, (12 copies)]/***aat***[*ble*, *tef1*p-*aat*-*pgk1*t) (An3HP9/*aat2*: 5 copies)]	This work
An3HP9/*pyc*2, 5, or 6	**3HP**[*gpdA*p*-panD-elf3*t*-bapat-eno*p*, gpdA*p*-hpdh-trpC*t*, ptrA*, (12 copies)]/***pyc***[*ble*, *mbf1*p-*pyc*-*pgk1*t (An3HP9/*pyc*2: 8 copies)]	This work
An3HP9/*pyc*2/*oahA*Δ	**3HP**[*gpdA*p*-panD-elf3*t*-bapat-eno*p*, gpdA*p*-hpdh-trpC*t*, ptrA*, (12 copies)]/***pyc***[*ble*, *mbf1*p-*pyc*-*pgk1*t (An3HP9/*pyc*2: 8 copies)]/***oahA***Δ(*oahA*::*hph*)	This work
An3HP9/*pyc*2/*ald6a*Δ	**3HP**[*gpdA*p*-panD-elf3*t*-bapat-eno*p*, gpdA*p*-hpdh-trpC*t*, ptrA*, (12 copies)]/***pyc***[*ble*, *mbf1*p-*pyc*-*pgk1*t (An3HP9/*pyc*2: 8 copies)]/***ald6a***Δ(*ald6a*::*hph*)	This work
An3HP9/*pyc*2/*ald6b*Δ	**3HP**(*gpdA*p*-panD-elf3*t*-bapat-eno*p*, gpdA*p*-hpdh-trpC*t*, ptrA*, (12 copies)]/***pyc***[/*ble*, *mbf1*p-*pyc*-*pgk1*t (An3HP9/*pyc*2: 8 copies)]// ***ald6b***Δ*(ald6b*::*hph*)	This work
An3HP9/*pyc*2/*ald3*Δ	**3HP**(*gpdA*p*-panD-elf3*t*-bapat-eno*p*, gpdA*p*-hpdh-trpC*t*, ptrA*, (12 copies)]/***pyc***[/*ble*, *mbf1*p-*pyc*-*pgk1*t (An3HP9/*pyc*2: 8 copies)]/***ald3***Δ*(ald3*::*hph*)	This work
An3HP9/*pyc*2/*uga2*Δ	**3HP**[*gpdA*p*-panD-elf3*t*-bapat-eno*p*, gpdA*p*-hpdh-trpC*t*, ptrA*, (12 copies)]/***pyc***[*ble*, *mbf1*p-*pyc*-*pgk1*t (An3HP9/*pyc*2: 8 copies)]//**uga2**Δ(*uga2*::*hph*)	This work
An3HP9/*pyc*2/*mct1*	3HP[*gpdA*p*-panD-elf3*t*-bapat-eno*p*, gpdA*p*-hpdh-trpC*t*, ptrA*, (12 copies)]*pyc*[/*ble*, *mbf1*p-*pyc*-*pgk1*t (An3HP9/*pyc*2: 8 copies)]/*mct1*(*pmbfA*p-*mct1*-*msf*t, *nat1*)	This work
An3HP9/*pyc*2/*ald6a*Δ*/*3HP-6	3HP(*gpdA*p*-panD-elf3*t*-bapat-eno*p*, gpdA*p*-hpdh-trpC*t*, ptrA*, (12 copies)]/*pyc*[*ble*, *mbf1*p-*pyc*-*pgk1*t (An3HP9/*pyc*2: 8 copies)]/*ald6*Δ(*ald6a*::*hph*)*/*3HP[*ubi4*p*-panD-elf3*t*-bapat-ubiSp, mbfA*p*-hpdh-trpC*t*,* loxP-*nptII* (15copies)]	This work

*Copy number estimated by Southern blotting analysis; **copy numbers estimated by whole genomic DNA sequencing

### Preparation of transgene expression constructs for gene overexpression or gene disruption in *A. pseudoterreus* and *A. niger*

In our previous study, the β-alanine pathway transgene expression cassette with pyrithiamine resistance gene (*ptrA*) of *Aspergillus oryzae* as a selection marker was described previously [28]. In this study, all transgene expression cassettes were prepared with Gibson assembly master mix (NEB, Ipswich, MA, USA) and the DNA fragments were isolated by PCR with Phusion high-fidelity DNA polymerase (Thermo Fisher Scientific, Waltham, MA 02451, USA). Different transgene expression cassettes, related intermediate plasmids, or selection marker gene cassettes prepared for this study are thoroughly described in the section of “Detailed description of transgene vector construction for selected gene overexpression or disruption in *Aspergillus pseudoterreus* or *Aspergillus niger*” in Additional file 1.

### Culture methods

Pyrex 125 ml or 250 ml glass Erlenmeyer flasks were prepared by filling with 5% Contrad 70 (Decon Labs, Inc., King of Prussia, PA, USA) and soaked overnight to remove any potential residues on the inside surface of flasks prior to general dishwashing. Silicon sponge closures were used for all flask cultures. The biomass of transgenic clones and parent strain for genomic DNA isolation were prepared from 2 mL stationary CM cultures with proper antibiotics and grown in 13 × 100 mm glass culture-tubes for 24–36 h at 30 °C. The biomass formed on the surface of the liquid culture medium was collected, frozen immediately in liquid nitrogen and dried in the VirTis benchtop manifold freeze dryer (SP Scientific, Gardiner, NY, USA). For 3-HP production, 35 ml of citric acid production (CAP) medium was prepared by the following previous descriptions [31]. Production medium B (RDM) [33] or modified production medium B (mRDM) [28, 33] that contains 20 × TE (trace elements: 4 mg/l CuSO_4_.5H_2_O; 110 mg/l FeSO_4_.7H_2_O; 14 mg/l MnCl_2_.4H_2_O; and 26 mg/l ZnSO_4_.7H_2_O) was also used. The fermented sugars liberated from corn stover by deacetylated and disk-refined process (DDR), in which the biomass was deacetylated with dilute alkaline at low temperature first, then mechanically refined in an industrial size disk refiner, and finally enzymatically hydrolyzed [DDR-EH; Batch 1–19-05, 20190829, [57]], were obtained from the Pilot Plant at National Renewable Energy Laboratory (Golden, CO, USA).

### Chemical-mediated protoplast transformation of *A. niger*

The protoplast preparation and chemical-mediated transformation followed the method described by Dai et al. [58] for *A. niger*. Briefly, the 14.4 kb plasmid DNA of the β-alanine pathway transgene expression construct was linearized by restriction enzyme *Eco*RV and concentrated down to about 1 µg/µl with Microcon-30 kDa centrifugal filter unit (MilliporeSigma, Burlington, MA, USA). Ten microliters of the linearized plasmid DNA were used for protoplast transformation in *A. niger*. For transgene overexpression of *A. niger aat1*, *pyc*, the *aat1*-*pyc*, or *mct1* gene in *A. niger*, about 3–5 µg of linearized plasmid DNAs by proper restriction enzymes were used for protoplast transformation. For the gene deletion construct of *ald6a*, *ald6b*, *oah1*, or *uga2* gene homolog, about 1 µg of linearized plasmid DNAs by restriction enzyme *Pme*I was used for protoplast transformation in *A. niger*. Usually, about 5 to 12 transformed clones were picked randomly for the evaluation of 3-HP production and the effects of selected genes on 3-HP production. The chemical-mediated protoplast transformation of *A. pseudoterreus* was mainly followed the previous description [59].

### Total genomic DNA isolation for PCR, Southern blotting analysis, and short-read whole genomic DNA sequencing

Total genomic DNA was isolated from *A. niger* or *A. pseudoterreus* cells using a cetyltrimethylammonium bromide (CTAB) extraction method with some modifications. Briefly, 50–100 mg of lyophilized biomass and two 3.5 mm diameter glass beads were transferred into a 2 mL polypropylene micro-vial, where biomass was pulverized into fine power with a Mini-Beadbeater-8 (Bio Spec Products Inc., Bartlesville, OK, USA) for 50 s. The disrupted cells in microcentrifuge tubes were re-suspended with 800–900 µl of CTAB solution and incubated at 60 °C for 30 ~ 45 min and inverted occasionally. The genomic DNA in the supernatant of the cell extracts was extracted with 300 µl of phenol/chloroform solution and precipitated with 1 volume of 2-propanol. The genomic DNA was resuspended with 200 µl of 50TE (50 mM Tris–HCl, pH8.0 and 10 mM EDTA, pH8.0) and 25 µg of RNase and incubated for 30 ~ 45 min at 50 °C. After RNase treatment, the genomic DNA was extracted twice with 125 µl of phenol/chloroform solution and once with chloroform. The genomic DNA in the supernatants was precipitated with 1 M NaCl and 2 volume of 95% ethanol for 15 min at room temperature and centrifugation at 10,000 × g for 8 min. Finally, the genomic DNA pellet was washed with 70% ethanol and was resuspended in 10 mM Tris–HCl (pH 8.0) buffer at 50 °C for 15–20 min and the concentration was determined with a Qubit fluorometer (Thermo Fisher Scientific, Waltham, MA, USA). Fifty to seventy ng of total genomic DNA were used for PCR analyses.

For Southern blotting analyses of heterologous expression of β-alanine pathway in either *A. niger* or *A. pseudoterreus*, 1 µg of total genomics DNA was digested with the restriction endonuclease *Bam*HI, *Eco*RV, or *Hin*dIII. The genomic DNA fragments were separated in 1% agarose gel electrophoretically and transferred onto the Hybond-N^+^ nylon membrane (GE Healthcare Bio-Sciences, Pittsburgh, PA, USA) with alkaline capillary transfer method. The 1.0 kb 3’-end of genomic DNA fragments of *A. pseudoterreus cad1* gene was used for the preparation of the biotin-labeled probe. The genomic DNA in the Hybond-N^+^ nylon membrane was hybridized with the biotin-labeled probe overnight at 60 °C in the Problot Hybridization Oven (Labnet International, Edison, NJ, USA). The genomic DNA on the hybridized membrane was visualized with North2South chemiluminescent detection kit (Pierce Protein Research Products, Rockford, IL, USA) in Analytikjena UVP ChemStudio (Analytik Jena US, Upland, CA, USA).

The short-read whole genomic DNA sequencing was carried out by Azenta Life Sciences (South Plainfield, NJ, USA). The integration copy number was estimated by fold-increase of reads mapped to the expression construct versus background single copy regions of the genome. The sequenced short-reads were mapped to the reference genome sequence of *A. niger* ATCC 1015 (https://mycocosm.jgi.doe.gov/Aspni7/Aspni7.home.html) augmented with the overexpressed gene sequence using BWA-MEM [60]. The mapped reads were sorted using SAMtools [61] and duplicate reads were marked using Picard Toolkit (https://github.com/broadinstitute/picard#citing) to produce BAM files for copy number estimation. The copy numbers of β-alanine pathway genes and engineered native genes were estimated using CNVnator [62]. The mapped reads were counted using bin sizes of 100, 200, and 1,000 bp, and the read depth signal was partitioned into segments for each bin size. The average and standard deviation of read depth signal were evaluated for bin sizes of 100 and 200 bp, and copy number genotype was estimated based on the normalized read depth using the bin size of 100 bp.

### Metabolites analysis by HPLC

The extracellular metabolites were quantified by HPLC. Twenty-five microliters of the samples filtered with 0.2 µm syringe filters were analyzed for 45 min using an Aminex HPX-87H ion exclusion column with a 1 mM H_2_SO_4_ flow of 0.6 ml/ml. The temperature of the column was 60 °C. The refractive index at 45 °C and the UV absorption at 210 nm were measured.

### Sample preparation for metabolomics and proteomics analyses

Briefly, the culture supernatants or biomass (cell pellet) for *A. niger* or *A. pseudoterreus* were harvested at day 4. For quantification of extracellular metabolites diluted spent medium samples (by a 1/8 factor) were dried, prepared, and analyzed as described previously [28]. The cell pellets were extracted using the MPLex protocol [63] and extracts were analyzed using GC–MS as explained previously in detail [64]. The protein interlayer pellet was digested and prepared for global proteomics analysis and targeted proteomics analysis, the latter using heavy labeled peptides. Instrument acquisition and data analysis were done as described in a previous publication [28]. Global proteomics data were generated using a Q Exactive Plus mass spectrometer (Thermo Fisher Scientific, Waltham, MA, USA) in data-dependent acquisition mode.

## Supplementary Information

**Additional file 1: Detailed Description of transgene vector construction for gene overexpression or disruption in *A. pseudoterreus* or *A. niger*.** The vector prepared are: **3HP4025**, **3HP4028**, **3HP4069**, **3HP4070**, **3HP4071**, **3HP4074**, **3HP4076**, **3HP4077**, **3HP4102, 3HP4103**, **3HP4104**, **3HP4108**, **3HP4109**, **3HP4114**, **3HP4126**, **3HP4134**, **3HP4136**, **3HP4140**, **3HP4144**, and **3HP4145**. **Table S1**. Oligos used for transgene vector constructions of the gene overexpressions or disruptions. **Figure S1.** The diagram of the β-alanine 3HP pathway transgene expression cassette with *A. pseudoterreus cad1* gene locus targeting and Southern blotting analyses of transgenic *A. pseudoterreus.* (A) the diagram of the β-alanine 3HP pathway (**3HP4028**, **Ap3HP**); (B) the diagram of the β-alanine 3HP pathway with the pattern of restriction endonuclease *Bam*HI, *Eco*RV, and *Hin*dIII; (C) the restriction fragment length polymorphism of *Bam*HI or *Eco*RV in selected transgenic strains; (D) the restriction fragment length polymorphism of *Hin*dIII in selected transgenic strains. **Figure S2.** 3-hydroxypropionic acid and itaconic acid production in the selected individual transgenic strains of *A. pseudoterreus* with overexpression of the β-alanine 3HP pathway transgene expression cassette in the modified RDM medium at 30 °C and 200 rpm for 7 days. **Figure S3.** The diagram of β-alanine 3HP pathway transgene expression cassette with two identical copies of β-alanine 3HP pathway (**3HP4046, 2 × 3HP**). **Figure S4.** The diagram of β-alanine 3HP pathway transgene expression cassette with E. coli hygromycin B phosphotransferase (hph) marker gene (**3HP4070**). **Figure S5.** The diagram of β-alanine 3HP pathway along with an additional *aat1* transgene overexpression under the control of *A. pseudoterreus tef1* gene promoter (**3HP4071**). **Figure S6.** Southern blot analysis confirmed the β-alanine 3HP pathway random integrations into the chromosomes of *A. nige*r in the single spore isolates of transgenic strain An3HP5, An3HP9, An3HP10, and An2 × 3HP1 with multiple copies of chromosomal insertions. (A). restriction map of the plasmid DNA fragments containing the β-alanine 3HP pathway used for random integration with restriction endonucleases of *Bam*HI (B), or *Hin*dIII (C). **Figure S7.** The diagram of transgene overexpression cassettes of *A. niger* aspartate aminotransferase (*aat1*), pyruvate carboxylase (*pyc*) or their combination; (A). the *ble*, the bacterial bleomycin resistance gene; *Tef1*P, *A. niger tef1* gene promoter; *A. niger aat1*, aspartate aminotransferase; T*pgk*, *A. niger pgk* transcriptional terminator (**3HP4074**); (B). & (C) *mbf1*P, *A. niger mbf1* gene promoter; *pyc*, *A. niger pyc* gene without *aat1* (B, **3HP4076)** & with *aat1* (C, **3HP4077**); (D). the *nat1* (*Streptomyces noursei* nourseothricin N-acetyl transferase optimized for the codon usage of *Saccharomyces cerevisiae*) selection marker under the control of *A. nidulans trpC* promoter and *A. niger trpC* transcriptional terminator (**3HP4114**); (E) the *nat*, nourseothricin N-acetyl transferase marker gene; the *mct1*, *A. niger* monocarboxylate transporter; the T*mct1*, *A. niger mct1* transcriptional terminator (**3HP4126**). **Figure S8.** 3-hydroxypropionic acid production in the selected individual transgenic strains of *A. niger* with overexpression of cytosolic aspartate aminotransferase (*aat*, A), pyruvate carboxylase (*pyc*, B), or aspartate aminotransferase + pyruvate carboxylase (*aat*-*pyc*, C) in An3HP9 strain grown in modified RDM medium at 30 °C and 200 rpm for 7 days. **Figure S9.** The diagram of gene disruption constructs of *A. niger ald6a*, *ald6b*, *ald3*, *uga2*, or *oahA* gene. (A). *5’-oahA* and *3’-oahA* are upstream and downstream fragments of *oahA* gene (**3HP4102**); (B). *5’-ald6a* and *3’-ald6a* are fragments of upstream and downstream of *ald6a* gene (**3HP4103**); (C). *5’-ald6b* and *3’-ald6b* are fragments of upstream and downstream of *ald6b* gene (**3HP4104**); (D). *5’-ald3* and *3’-ald3* are fragments of upstream and downstream of *ald3* gene (**3HP4108**); and (E). *5’-uga2* and *3’-uga2* are upstream and downstream fragments of *uga2* gene (**3HP4109**). **Figure S10.** The diagram of new β-alanine 3HP pathway transgene expression cassette (**3HP4145)** with loxP-*nptII* marker gene recycle for *A. niger*. The Tet-On/Cre-loxP system (**3HP4140**) for marker gene recycle conditionally activated by doxycycline, *nat1*, *S. noursei* nourseothricin acetyltransferase (resistance) marker gene; *ubi1S27*p, *A. niger ubi1S27* promoter; rtTA2A, the reverse tetracycline transactivator; TetO7, tetracycline resistance operon; Pmn, *A. nidulans gpdA* minimal promoter; Cre, Cre recombinase; Ac*trpC*t, *A. carbonarius trpC* transcriptional terminator; (**B**). the bacterial neomycin-resistance (*nptII*) marker gene under the control of *A. niger* malate dehydrogenase (*mdh*p) promoter and *A. nidulans trpC* transcriptional terminator (*trpC*t) (**3HP4134**); (**C**). backbone marker gene cassette with 31 bp loxP fragments fused at 5’- and 3’-end of *nptII* marker gene (**3HP3136**); (**D**). the intermediate transgene expression cassette (**3HP4144**) contains the *ubi4*p, *A. niger ubi4* gene promoter; PAND, *T. castaneum* aspartate decarboxylase; *elf3*t, *A. niger* elongation factor 3 transcriptional terminator; BAPAT, *B. cereus* β-alanine-pyruvate aminotransferase; and *ubiS*p, *A. niger ubi1S* promoter; (**E**). the final new β-alanine 3HP pathway (**3HP4145**) was assembled with the HPDH, *E. coli* 3-hydroxypropionate dehydrogenase under the control of *A. niger mbfA* promoter (*mbfA*p) and *A. nidulans trpC* transcriptional terminator(*trpC*t). **Figure S11.** The effects of culture medium and pH on 3-HP production and spore germination in *A. niger* strain An3HP9 grown at 30 °C and 200 rpm for 7 days. (A) 3-HP and DCW titer and yield and (B) the percentage of spore germination.

## Data Availability

All data generated or analyzed during this study are included in this published article and its supplementary information file.

## Abbreviations

3-HP: 3-Hydroxypropionic acid
3HP: β-Alanine pathway for 3-hydroxypropionic acid production
PAND: Aspartate decarboxylase of *Tribolium castaneum*
BAPAT: β-Alanine-pyruvate aminotransferase of *Bacillus cereus*
HPDH: 3-Hydroxypropionate dehydrogenase of *Escherichia coli*
AAT: Aspartate aminotransferase
PYC: Pyruvate carboxylase
DBTL: Design-build-test-learn
CAP: Citric acid production medium
DDR-EH: Disk refining and enzymatic hydrolysis
RDM: Production medium B
mRDM: Modified production medium B
TE: Trace elements
CM: Complete medium
MM: Minimal medium
PDA: Potato dextrose agar
*hph*: *E. coli* Hygromycin B phosphotransferase marker gene
*cad1*: Cis-aconitate decarboxylase
*gpdA*: Glyceraldehyde-3-phosphate dehydrogenase
*eno1*: Alpha-enolase
*elf3*: Elongation factor 3
*trpC*: Tryptophan C gene
*ald6*: Malonate semialdehyde dehydrogenase gene 6
*ald3*: Malonate semialdehyde dehydrogenase gene 3
*uga2*: Succinate semialdehyde dehydrogenase
*oahA*: Oxaloacetate hydrolase
*mct1*: Monocarboxylate transporter
*nat1*: *Streptomyces noursei* Nourseothricin N-acetyl transferase
*nptII*: Bacterial neomycin-resistance (*nptII*) marker gene
