# Supplementary material for: Metabolic engineering to improve production of 3-hydroxypropionic acid from corn-stover hydrolysate in Aspergillus species
Source: Biotechnol Biofuels Bioprod. 2023 Mar 29;16:53. doi: 10.1186/s13068-023-02288-1 (PMC10061894; doi:10.1186/s13068-023-02288-1)
Supplement: Supplementary file 1 — Additional file 1: Detailed Description of transgene vector construction for gene overexpression or disruption in A. pseudoterreus or A. niger. The vector prepared are: 3HP4025, 3HP4028, 3HP4069, 3HP4070, 3HP4071, 3HP4074, 3HP4076, 3HP4077, 3HP4102, 3HP4103, 3HP4104, 3HP4108, 3HP4109, 3HP4114, 3HP4126, 3HP4134, 3HP4136, 3HP4140, 3HP4144, and 3HP4145. Table S1. Oligos used for transgene vector constructions of the gene overexpressions or disruptions. Figure S1. The diagram of the β-alanine 3HP pathway transgene expression cassette with A. pseudoterreus cad1 gene locus targeting and Southern blotting analyses of transgenic A. pseudoterreus. (A) the diagram of the β-alanine 3HP pathway (3HP4028, Ap3HP); (B) the diagram of the β-alanine 3HP pathway with the pattern of restriction endonuclease BamHI, EcoRV, and HindIII; (C) the restriction fragment length polymorphism of BamHI or EcoRV in selected transgenic strains; (D) the restriction fragment length polymorphism of HindIII in selected transgenic strains. Figure S2. 3-hydroxypropionic acid and itaconic acid production in the selected individual transgenic strains of A. pseudoterreus with overexpression of the β-alanine 3HP pathway transgene expression cassette in the modified RDM medium at 30 °C and 200 rpm for 7 days. Figure S3. The diagram of β-alanine 3HP pathway transgene expression cassette with two identical copies of β-alanine 3HP pathway (3HP4046, 2 × 3HP). Figure S4. The diagram of β-alanine 3HP pathway transgene expression cassette with E. coli hygromycin B phosphotransferase (hph) marker gene (3HP4070). Figure S5. The diagram of β-alanine 3HP pathway along with an additional aat1 transgene overexpression under the control of A. pseudoterreus tef1 gene promoter (3HP4071). Figure S6. Southern blot analysis confirmed the β-alanine 3HP pathway random integrations into the chromosomes of A. niger in the single spore isolates of transgenic strain An3HP5, An3HP9, An3HP10, and An2 × 3HP1 with multiple copies of chro [file 13068_2023_2288_MOESM1_ESM.docx]

**Detailed description of transgene vector construction for gene overexpression or disruption in *Aspergillus pseudoterreus* or *Aspergillus niger*.**

All transgene over-expression or gene disruption constructs were prepared by Gibson assembly method (reference, Ipswich, MA, USA 01938) with DNA fragments isolated by PCR from either related plasmid or genomic DNAs of *Aspergillus carbonarius*, *A. niger*, *Aspergillus oryzae*, or *A. pseudoterreus* with proper oligo pairs and Phusion high-fidelity DNA polymerase (Thermo Fisher Scientific, Waltham, MA, USA 02451). The first β-alanine pathway transgene expression cassette with pyrithiamine resistance gene (*ptrA*; Genbank: AF217503) of *A. oryzae* as a selection marker shown in supplementary **Figure S1A** was prepared as follows: the first 987bp DNA fragment of *cad1* (Genbank: UDM55691.1, ***5’-cad1***) gene upstream region containing 535bp coding sequence of N-terminus was isolated by PCR with oligo pair 1969cad1/1670cad1 (**Table S1**); the second 813bp DNA fragment of *A. niger* *gpdA* (Genbank: XP_025477631, ***gpdA*p**) promoter with oligo pair 1971gpdA1/1972gpdA2; the third 1623bp DNA fragment of aspartate decarboxylase (*Tribolium castaneum panD*, **PAND**) with 1973pan1/1974pan2 and the synthetic plasmid DNA of codon usage optimization for *A. pseudoterreus* as a template; the forth 1040bp DNA fragment of bi-direction transcriptional terminator of *A. niger* elf3-multifunctional chaperone (*elf3*, Genbank: CAK43351.1, ***elf3*t**) with oligo pair 1975ter1/1976ter2; the fifth 1350bp reverse complement DNA fragment of β-alanine-pyruvate aminotransferase (*Bacillus cereus* *bapat*, **BAPAT**) with oligo pair 1977bap1/1978bap2 and the synthetic plasmid DNA of codon usage optimization for *A. pseudoterreus* as a template; and final 704bp complement DNA fragment of *A. niger* enolase (Genbank: AM270411.1, ***eno1*p**) promoter with oligo pair 1979eno1/1980eno2-HpaI. All 6 DNA fragments were assembled together into the pBSK(-) vector linearized with restriction endonuclease *Hin*dIII and *Pst*I to form new intermediate vector **3HP4025**. The 885bp DNA fragment of *Aspergillus nidulans* *gpdA* promoter was prepared by PCR with oligo pair 1981gpdA1/1982gpdA2 and pAN7-1 plasmid DNA (Genbank: Z32698.1, ***gpdA*p**) as a template; the 744bp DNA fragment of 3-hydroxypropionate dehydrogenase (*Escherichia coli* *hpdh*, **HPDH**) with oligo pair 1983hpd1/1984hpd2 and the synthetic plasmid DNA of codon usage optimization for *A. pseudoterreus* as a template; the 473bp DNA fragment of *A. nidulans* *trpC* (***trpC*t**) transcriptional terminator with oligo pair 1985trp1/1986trp2 and pAN7-1 plasmid DNA as a template; the 2005bp DNA fragment of *A. oryzae* *ptrA* gene with oligo pair 1987ptrA1/1988ptrA2 (***ptrA***); and 908bp DNA fragment of *A. pseudoterreus* *cad1* gene downstream region containing 519bp coding sequence of its C-terminus and transcriptional terminator with oligo pair 1989cad3/1990cad4 (***3’-cad1***). Above 5 DNA fragments were assembled into the intermediate vector **3HP4025** linearized with the restriction endonuclease *Hpa*I, which was introduced into the **3HP4025** with oligo 1980eno2-HpaI, to form the entire β-alanine pathway transgene expression cassette **3HP4028**. The entire transgene expression construct was verified by Sanger DNA sequencing. The second β-alanine pathway transgene expression cassette was to construct two identical copies of the β-alanine pathway, where the plasmid **3HP4028** was linearized by restriction endonuclease *Eco*RI and assembled with the entire fragment of the β-alanine pathway, which was isolated by PCR with oligo pair 2085HP2F/2086HP2R and 3HP4028 as a DNA template to form the plasmid **3HP4046** (supplementary **Figure S3**).

The third β-alanine pathway transgene expression cassette **3HP4070** with *E.* *coli* hygromycin B phosphotransferase (*hph*) marker gene (supplementary **Figure S4**) was prepared with the plasmid **3HP4028** and pCB1003 (<http://www.fgsc.net/fgn41/carroll.html>) as the DNA templates for PCR as follows: the first 2759bp DNA fragment containing 326bp of 5’-end *cad1* coding sequence of *A. pseudoterreus*, 810bp of *A. niger* *gpdA* promoter, and 1623bp DNA fragment of *panD* with oligo pair 2201/2202; the second 3091bp DNA fragment containing the 1040bp DNA fragment of bi-direction transcriptional terminator of *A. niger* *elf3*, the 1350bp reverse complement DNA fragment of *bapat*, and 701bp reverse complement DNA fragment of *A. niger* *eno1* promoter with oligo pair 2203/2204. These two fragments were assembled into the pBSK(-) vector linearized with restriction endonuclease *Hin*dIII and *Pst*I to form new intermediate vector **3HP4069**. The 1360bp DNA fragment of *hph* marker gene was prepared by PCR with oligo pair 2213hphF/2214hphR and plasmid DNA pCB1003 as a template. The 2385bp DNA fragment containing 885bp DNA fragment of *A. nidulans* *gpdA* promoter, 744bp DNA fragment of *hpdh*, 473bp DNA fragment of *A. nidulans* *trpC* transcriptional terminator, and 283bp upstream region of *A. oryzae* *ptrA* gene was isolated with oligo pair 2215hpdF/2216hpdR. These two fragments were further assembled into the 3HP4069 linearized with restriction endonuclease *Xba*I to form the **3HP4070** transgene expression vector. The entire transgene expression construct was verified by Sanger DNA sequencing.

The fourth β-alanine pathway transgene expression cassette with an additional *A. pseudoterreus* aspartate aminotransferase gene (Ap*aat1*, jgi|Aspte1|7965|ATET_04402; supplementary **Figure S5**) was prepared by PCR with oligo pairs of PAptefF/PAptefR for 956bp *A. pseudoterreus* *tef1* gene (Ap*tef1*, jgi|Aspte1|9546|ATET_05983) promoter and the ApaatF/Apaat for 1898bp *A. pseudoterreus* aspartate aminotransferase entire coding region and 269bp its transcriptional terminator. The fragments were assembled into **3HP4070** linearized with restriction endonuclease *Xba*I to form transgene expression cassette **3HP4071**. The entire transgene expression construct was verified by Sanger DNA sequencing.

The PCR fragments for *A. niger* aspartate aminotransferase gene (*aat1*, GenBank: EHA22111) over-expression construct **3HP4074** (supplementary **Figure S7A**, *ble*, *tef1*P:*aat*:*pgk1*T) were prepared with the oligo pair bleF1/bleR1 and plasmid DNA pAN8-1 (GenBank: Z32751.1) for 1550bp DNA fragment of the bacterial bleomycin resistance (*ble*) marker gene, the oligo pair PAntef1F/PAntef1R for 870bp DNA fragment of translation elongation factor 1 (*tef1*, Genbank: CBJ23536.1) promoter of *A. niger*, the oligo pair AnaatF/AnaatR for the 1401bp *aat1* cDNA of *A. niger*, and the oligo pair TAnpgkF1/TAnpgkR1 for 772bp DNA fragment of the transcriptional terminator of phosphoglerate kinase (*pgk*, jgi|aspin7|1147902) gene of *A. niger*. All PCR fragments were assembled together into the pBSK(-) linearized by *Hin*dIII and *Pst*I restriction enzymes with Gibson assembly master mix (NEB, Ipswich, MA, USA). Similarly, the over-expression transgene construct for pyruvate carboxylase were prepared with the oligo pairs of bleF1/bleR2 (*ble*) for 1550bp DNA fragment of the *ble* gene, TAnpgkF2/TAnpgkR1 (T*pgk*) for 772bp DNA fragment of the transcriptional terminator of *pgk*, AnpycF/AnpycR for 3579bp cDNA fragment of *A. niger* pyruvate carboxylase cDNA (*pyc*; jgi|Aspni7|1031996), and PAnmbf1F/PAnmf1R for 1499bp DNA fragment of *A. niger* *mbf1* gene (jgi|Aspni7|1145066) promoter (supplementary **Figure S7B, 3HP4076**) or their combination, where *pyc* overexpression cassette was incorporated into the downstream of *aat1* overexpression cassette right after the TpgkF2/TpgkR1 (*pgk*t) DNA fragment (supplementary **Figure S7C, 3HP4077**). The entire transgene expression construct was verified by Sanger DNA sequencing.

Prior to building the transgene overexpression construct for *A. niger* monocarboxylate transporter (*mct1*, jgi|Aspni7|1163060), the new selection marker *nat1* (*Streptomyces noursei* nourseothricin N-acetyl transferase optimized for the codon usage of *Saccharomyces cerevisiae*) gene under the control of *A. nidulans* *trpC* promoter and *A. niger* *trpC* transcriptional terminator cassette was prepared. The DNA fragment for *A. nidulans* *trpC* promoter and *A. niger* *trpC* transcriptional terminator were isolated with the oligo pair 2555trpF1/2556trpR1 and 2559trpF2/2560trpR2 with the pCSN44 plasmid (GenBank: LT726870.1) as a template for *trpC* promoter and with *A. niger* genomic DNA as a template for *A. niger* *trpC* transcriptional terminator, respectively. The *nat1* coding region was obtained by PCR with oligo pair 2557nat1F/2558nat1R and the plasmid DNA CHCp9 (1) as a template. All three DNA fragments were assembled to form vector (supplementary **Figure S7D, 3HP4114**). The *mct1* overexpression construct was prepared with the oligo pairs of nat1F/nat1R for 1250bp DNA fragment of *nat1* marker gene with **3HP4114** as a template, PAnmbf1/PAnmbf1R for 1351bp DNA fragment of *A. niger* *mbf1* gene promoter and AnmctF/AnmctR for 1544bp DNA fragment of *A. niger* *mct* gene coding sequence and its transcriptional terminator, which were assembled into pBSK(-) linearized with *Hin*dIII/*Pst*I restriction endonucleases to form **3HP4126** (supplementary **Figure S7E**). The entire transgene expression construct was verified by Sanger DNA sequencing.

Gene disruption constructs for *oahA* (jgi/Aspni7/1145269), *ald6a* (jgi/Aspni7/1182225), *ald6b* (jgi/Aspni7/201822), *ald3* (jgi/Aspni7/1126238), and *uga2* (jgi/Aspni7/57046) from *A. niger* were prepared by Gibson assembly of related DNA fragments isolated via PCR with the following oligo pairs: 5AnoahAF/5AnoahAR, hphF1/hphR1, and 3AnoahAF/3AnoahAR for *oahA* gene (supplementary **Figure S9A**, **3HP4102**); 5Anald6aF/5Anald6haR, hphF2/hphR2, and 3Anald6aF/3Anald6aR for *ald6a* gene (supplementary **Figure S9B**, **3HP4103**); 5Anald6bF/5Anald6bR, hphF3/hphR3, and 3Anald6bF/3Anald6bR for *ald6b* gene (supplementary **Figure S9C**, **3HP4104**); 5Anald3F/5Anald3R, hphF4/hphR4, and 3Anald3F/3Anald3R for *ald3* gene (supplementary **Figure S9D**, **3HP4108**); 5Anuga2F/5Anuga2R, hphF5/hphR5, and 3uga2F/3uga2R for *uga2* gene (supplementary **Figure S9E**; **3HP4109**).

For further genetic modification in the transgenic strain, the marker recycling strategy was applied to overcome the limitation of selection marker genes in *A. niger*. The Tet-On/Cre-loxP system (2, 3) was incorporated into the new transgene expression or gene disruption. The Tet-On/Cre-loxP transgene expression construct was prepared with the oligo pairs of nat1F/nat1R for 1250bp DNA fragment of entire *nat1* marker gene with 3HP4114 as a template, ubi1F/ubi1R, rtTAF/rtTAR, tetO7F/tetO7R, and trpCF/trpCR for 848bp DNA fragment of *A. niger* *ubi1S27* gene (jgi|Aspni7|1135631) promoter, 1036bp DNA fragment of reverse tetracycline transactivator (rtTA2S), 1550bp DNA fragment of tetracycline resistance operon/*gpdA* mini promoter of *A. nidulans*/Cre recombinase (tetO7-Pmn-Cre), in which the rtTA2S and tetO7-Pmn-Cre were synthesized with codon usage optimization for *A. niger*, and 554bp DNA fragment of *A. carbonarius* *trpC* transcriptional terminator, respectively. All DNA fragments were assembled into the pBSK(-) linearized with *Hin*dIII/*Pst*I restriction endonucleases to form transgene expression vector **3HP4140** (supplementary **Figure S10A**). The entire transgene expression construct was verified by Sanger DNA sequencing.

The *nptII* [the neomycin phosphotransferase II gene, (4)] marker gene for *A. niger* was constructed with the DNA fragments isolated by PCR with oligo pair 2662mdhFp/2663mdhRp for 965bp DNA fragment of *A. niger* *mdh* (jgi|Aspni7|1143375) gene promoter and oligo pair 2664npt2F/2665npt2R for 1138 bp DNA fragment of *nptII* gene coding region (GenBank: AF485783.1, 795bp) and *A. nidulans* *trpC* transcriptional terminator (GenBank: U24705.1, 343bp). The DNA fragments were assembled into the pBSK(-) linearized with *Hin*dIII/*Pst*I restriction endonucleases to form plasmid vector **3HP4134** (supplementary **Figure S10B**). To introduce the 31bp loxP fragments into the *nptII* marker gene, the oligo pair loxPnpt2F/loxPnpt2R was used to isolate the *nptII* marker gene from **3HP4134** plasmid DNA and assembled into the pBSK(-) linearized with *Hin*dIII/*Pst*I restriction endonucleases to form plasmid vector **3HP4136** (supplementary **Figure S10C**).

To construct the fifth β-alanine 3HP pathway transgene expression cassette with loxP-*nptII* as a selection marker and a new set of *A. niger* promoters, the **3HP4028** plasmid DNA was used for a DNA template for isolation of *panD*, *bapat*, *hpdh*, *elf3t* and *trpCt* with the following oligo pairs: 2728pu4F/2729pu4R for 819bp DNA fragment of *A. niger* *ubi4* (jgi|Aspni7|1146681) gene promoter, 2730panF/2731panR for 2198bp DNA fragment of *panD* gene and a part of *elf3*t, 2732bapatF/2733bapatR for 1823bp DNA fragment of *bapat* gene and a part of *elf3*t, and 847bp DNA fragment of 2734pus1F/2735pus1R for *A. niger* *ubiS27* gene promoter in reverse complementation. The first 4 DNA fragments were assembled into the **3HP4136** vector linearized with restriction endonuclease *Xho*I to form plasmid **3HP4144** (supplementary **Figure S10D**). The oligo pair 2736pmbF/273pmbR for 1199bp DNA fragment of *A. niger* *mbfA* promoter, and 2738hpdhF/2739hpdhR for 1257bp DNA fragment of *hpdh* gene and *A. nidulans* *trpC* transcriptional terminator. These two fragments were assembled into the plasmid **3HP4144** linearized with restriction endonuclease *Xba*I to the final transgene expression construct **3HP4145** shown in supplementary **Figure S10E**. The entire transgene expression construct was verified by Sanger DNA sequencing.

1. Calvey CH, Willis LB, Jeffries TW. An optimized transformation protocol for Lipomyces starkeyi. Curr Genet. 2014;60(3):223-30.

2. Jiang B, Zhang R, Feng D, Wang F, Liu K, Jiang Y, et al. A Tet-on and Cre-lox P based genetic engineering system for convenient recycling of selection markers in Penicillium oxalicum. Front Microbiol. 2016;7:485.

3. Meyer V, Wanka F, van Gent J, Arentshorst M, van den Hondel CA, Ram AF. Fungal gene expression on demand: an inducible, tunable, and metabolism-independent expression system for Aspergillus niger. Appl Environ Microbiol. 2011;77(9):2975-83.

4. Beck E, Ludwig G, Auerswald EA, Reiss B, Schaller H. Nucleotide sequence and exact localization of the neomycin phosphotransferase gene from transposon Tn5. Gene. 1982;19(3):327-36.

**Table S1**: Oligos used for transgene vector constructions of selected gene over-expressions or disruptions

|  | **β-alanine pathway transgene expression cassette with *ptrA* marker gene**  **(plasmid vector 3HP4028)** |
| --- | --- |
| 1969cad1 | ccctcgaggtcgacggtatcgataGATATCGGTTGTAGCAGCGTAAACAC |
| 1970cad2 | tctttcatagtagCCTTGGTGAACATCTTGAGG |
| 1971gpdA1 | atgttcaccaaggCTACTATGAAAGACCGCGATG |
| 1972gpdA2 | cgccggtggcgggCATTGTTTAGATGTGTCTATGTG |
| 1973pan1 | catctaaacaatgCCCGCCACCGGCGAGGACCA |
| 1974pan2 | atccaacccatcaGAGGTCGGAGCCCAGGCGTTCG |
| 1975ter1 | gggctccgacctcTGATGGGTTGGATGACGATG |
| 1976ter2 | tctggcccagctcTGAGTCCTAGATGGGTGGTG |
| 1977bap1 | catctaggactcaGAGCTGGGCCAGACATTCCTTC |
| 1978bap2 | gtccatcaacatgGAACTGATGATCGTCCAGGTCAC |
| 1979eno1 | cgatcatcagttcCATGTTGATGGACTGGAGGG |
| 1980eno2-HpaI | gaactagtggatcccccgggctgc**GttaaC**TCGAGCTTACAAGAAGTAGCC  GGCTACTTCTTGTAAGCTCGA**GttaaC**gcagcccgggggatccactagttc |
| 1981tdhA1 | acaggctacttcttgtaagctcgagttTCTGTACAGTGACCGGTGAC |
| 1982tdhA2 | tgaccagcacgatCATGGTGATGTCTGCTCAAG  C TTGAGCAGACATCACCATGatcgtgctggtca |
| 1983hpd1 | agacatcaccatgATCGTGCTGGTCACGGGCGC |
| 1984hpd2 | gccatcggtcctaTTGGCGGTGGACGTTCAGGC  GCCTGAACGTCCACCGCCAAtaggaccgatggc |
| 1985trp1 | cgtccaccgccaaTAGGACCGATGGCTGTGTAG |
| 1986trp2 | cccgtctgtcagaGAGCGGATTCCTCAGTCTCG  CGAGACTGAGGAATCCGCTCtctgacagacggg |
| 1987ptrA1 | gaggaatccgctcTCTGACAGACGGGCAATTGATTAC |
| 1988ptrA2 | gaatgttgctgagGAGCCGCTCTTGCATCTTTG  CAAAGATGCAAGAGCGGCTCctcagcaacattc |
| 1989cad3 | gcaagagcggctcCTCAGCAACATTCGCCATGTTC |
| 1990cad4 | actaaagggaacaaaagctggagctCAGCTCCACTGCTCATAGTCTTTG |
|  |  |
|  | **Two copies of β-alanine pathway transgene expression cassette with *ptrA* marker gene (plasmid vector 3HP4046, 2x3HP)** |
| 2085HP2F | acacaattctctatctcagatttgCTACTATGAAAGACCGCGATGGGC |
| 2086HP2R | ttcagtttcgtccgaggacttttggAATTCAAATCTGAGATAGAGAATTG |
|  |  |
|  | **β-alanine pathway transgene expression cassette with *hph* marker gene**  **(plasmid vector 3HP4070)** |
| 2201 | cgaggtcgacggtatcgataACGGATCGGCAAAGCAATCTACG |
| 2202 | atccaacccaTCAGAGGTCGGAGCCCAGGC |
| 2203 | cgacctctgaTGGGTTGGATGACGATGACTTC |
| 2212 | agtggatcccccgggctgcaCTCGAGCTTACAAGAAGTAGCCTG |
| 2213hphF | gcccgggggatccactagttGCTGGAGCTAGTGGAGGTCAAC |
| 2214hphR | ctgtacagagCGGTCGGCATCTACTCTATTC |
| 2215hpdF | atgccgaccgCTCTGTACAGTGACCGGTGAC |
| 2216hpdR-XbaI | agggaacaaaagctggagcttctAGAGAATTGTGTGGGATGAG |
|  |  |
|  | **β-alanine pathway transgene expression cassette with *hph* marker gene and *aat1* (plasmid vector 3HP4071)** |
| PAptefF | ctctcatcccacacaattctGAGCATCATCCCATGATAGC |
| PAptefR | aaaggggcgaACTGTTGTAGAAGATATCCGTTAG |
| ApaatF | ctacaacagtTCGCCCCTTTCCTCCTCTTC |
| ApaatR | gggaacaaaagctggagcttctagaCGAGCAATACGGAAGCGAATATC |
|  |  |
|  | **Over-expression of *A. niger* *aat* (plasmid vector 3HP4074)** |
| bleF1 | ctcgaggtcgacggtatcgataGtttaaaCTGAGGTGCAGTGGATGATTATTAATC |
| bleR1 | gaggtcaacgTTGATCTGCTTGATCTCGTC |
| PAntef1F | agcagatcaaCGTTGACCTCACAGGGATTTC |
| PAntef1R | aaaggggcgaCTTACTGTTGTAGAAGATATCCGTTAG |
| Anaat1F | caacagtaagTCGCCCCTTTCCTCCTCTTC |
| Anaat1R | gacagggcagTTATGAAGTCTCCCGAACTACGC |
| TAnpgk1F | gacttcataaCTGCCCTGTCGAGTAAGTAAATTTG |
| TAnpgk1R | actagtggatcccccgggctgcaagcttACTACAGAGAGGAGCTGAAG |
|  |  |
|  | **Over-expression of *A. niger* *pyc* (plasmid vector 3HP4076)** |
| bleR2 | atggccactgTTGATCTGCTTGATCTCGTCTC |
| 2271TAnpgkF2 | caaggcctagCTGCCCTGTCGAGTAAGTAAATTTG |
| AnpycF | TCttcagctcctctctgtagtaCTAGGCCTTGACGATCTTGCAGACAAGATCCTG |
| AnpycR | cttcaaaatgGCTGCTCCCCGCCAGCCCGA |
| PAnmbf1F | ggggagcagcCATTTTGAAGATGGATGAGAAGTC |
| PAnmbf1R | CTccaccgcggtggcggccgctGTTTAAACAGTGGCCATGAAATCCAATC |
|  |  |
|  | ***Nat1* selection marker gene construct (plasmid vector 3HP4114)** |
| 2555trpF1 | cgaggtcgacggtatcgataCAGAAGATGATATTGAAGGAGC |
| 2556trpR1 | aagtagtcatTTGGATGCTTGGGTAGAATAG |
| 2557nat1F | aagcatccaaATGACTACTTTGGATGACACTG |
| 2558nat1R | aaagctagagTTATGGACATGGCATGGACATG |
| 2559trpF2 | atgtccataaCTCTAGCTTTGTATTGTCTTTAAATTTAC |
| 2560trpR2 | agtggatcccccgggctgcaGTAAGTAGAAAGCTTTGGGG |
|  |  |
|  | ***mct1* gene overexpression (****plasmid vector 3HP4126)** |
| nat1F | cgaggtcgacggtatcgataACAGAAGATGATATTGAAGGAGC |
| nat1R | aacgaagaggCAGTAAGTAGAAAGCTTTGGG |
| PAnmbf1F | ctacttactgCCTCTTCGTTTCTGTGATGC |
| PAnmbf1R | cggtcgtatgCATTTTGAAGATGGATGAGAAGTC |
| AnmctF | cttcaaaatgCATACGACCGAGAAGATACC |
| AnmctR | agtggatcccccgggctgcaGGTTCTCTCCTTGCAGCAAAG |
|  |  |
|  | ***oahA* gene deletion (plasmid vector 3HP4102)** |
| 5AnoahAF | gaggtcgacggtatcgata**agctt**CTCAGCTGGGTGAAGAACAAC |
| 5AnoahAR | tagctccagcGTGATAGTGTTGGTCATGCTG |
| hphF1 | acactatcacGCTGGAGCTAGTGGAGGTCAAC |
| hphR1 | cgttagtatgCGGTCGGCATCTACTCTATTCC |
| 3AnoahAF | atgccgaccgCATACTAACGGAAGGGTCAG |
| 3AnoahAR | agtggatcccccgggctgcaGTACCACGCAAGCTTCGATATG |
|  |  |
|  | ***ald6a* gene deletion (plasmid vector 3HP4103)** |
| 5Anald6aF | gaggtcgacggtatcgatatctaGAGTAAACTGGTGCAGCTATC |
| 5Anald6aR | tagctccagcAGGTGAGGATGAGGAGAGCTTAG |
| hphF2 | atcctcacctGCTGGAGCTAGTGGAGGTCAAC |
| hphR2 | gactgtcttgCGGTCGGCATCTACTCTATTCC |
| 3Anald6aF | atgccgaccgCAAGACAGTCGATTTCATCCTC |
| 3Anald6aR | agtggatcccccgggctgcaCACTTGTCCAGACCGAGGTAC |
|  |  |
|  | ***ald6b* gene deletion (plasmid vector 3HP4104)** |
| 5Anald6bF | gaggtcgacggtatcgataGAGGATATGATCTCTTCAACTATAC |
| 5Anald6bR | tagctccagcTCTCAGGAGACATCTGTCTC |
| hphF3 | tctcctgagaGCTGGAGCTAGTGGAGGTCAAC |
| hphR3 | ctaccatcgaCGGTCGGCATCTACTCTATTCC |
| 3Anald6bF | atgccgaccgTCGATGGTAGTAGGATGTGG |
| 3Anald6bR | agtggatcccccgggctgcaCAGAGAAGGTGCAGATTGTG |
|  |  |
|  | ***aldh3* gene deletion (plasmid vector 3HP4108)** |
| 5Anald3F | cgaggtcgacggtatcgataGCTGGCGGTAGAAGGATTTTC |
| 5Anald3R | tagctccagcAATTGGCTGACGAAGAGTATAG |
| hphF4 | tcagccaattGCTGGAGCTAGTGGAGGTC |
| hphR4 | actgaagacgCGGTCGGCATCTACTCTATTC |
| 3Anald3F | atgccgaccgCGTCTTCAGTGATGCCGATATC |
| 3Anald3R | agtggatcccccgggctgcaCGTTTGGGCTTTGTCCTTTAG |
|  |  |
|  | ***uga2* gene deletion (plasmid vector 3HP4109)** |
| 5Anuga2F | cgaggtcgacggtatcgataCCAACTGGCGCTGTATAGATC |
| 5Anuga2R | tagctccagcGGTCTGAAAGTAGTCCTGTG |
| hphF5 | ctttcagaccGCTGGAGCTAGTGGAGGTC |
| hphR5 | acgaggattgCGGTCGGCATCTACTCTATTC |
| 5Anuga2F | atgccgaccgCAATCCTCGTCGACCATCAAAAAG |
| 5Anuga2R | agtggatcccccgggctgcaAGAGGGTATGAAGGAGGAG |
|  |  |
|  | **rtTA2S-TetO7-Cre system (plasmid vector 3HP4140)** |
| nat1F | aggtcgacggtatcgatatcACAGAAGATGATATTGAAGGAGC |
| nat1R | gatagggtggttgtctggatCAGTAAGTAGAAAGCTTTGGG |
| ubi1F | cgaggtcgacggtatcgataTCCAGACAACCACCCTATCTC |
| ubi1R | gtctggacatCTTGATGAAGGTCTGGGTTG |
| rtTAF | cttcatcaagATGTCCAGACTCGATAAGTC |
| rtTAR | cgtgatacgcTCCATGATTCATGACGTATATTC |
| tetO7F | gaatcatggaGCGTATCACGAGGCCCTTTC |
| tetO7R | gccgaagaccGATCCTCAATCACCATCCTCC |
| trpCF | attgaggatcGGTCTTCGGCTATAGTTCATTTTTATC |
| trpCR | agtggatcccccgggctgcaGTTGCGATCAGGTGTGTAATTG |
|  |  |
|  | ***mdh*P-neo marker gene (plasmid vector 3HP4134)** |
| 2662mdhFp | tcatcctcagGAACGACTCCAGAAGTGACTAAG |
| 2663mdhRp | gttcaatcatGGTGAAATTTGGGATTGTGAC |
| 2664npt2F | aaatttcaccATGATTGAACAAGATGGATTGC |
| 2665npt2R | agtggatcccccgggctgcaGACTCTGCTAAGCTATTCTTC |
|  |  |
|  | **LoxP-nptII vector (plasmid vector 3HP4136)** |
| loxPnpt2F | ggtcgacggtatcgataATAACTTCGTATAGCATACATTATACGAAGTTATGAACGACTCCAGAAGTGAC |
| loxPnpt2R | atcccccgggctgcaataacttcgtataatgtatgctatacgaagttatGACTCTGCTAAGCTATTCTTC |
|  |  |
|  | **New β-alanine 3-HP pathway** |
|  | **Intermediate vector (plasmid vector 3HP4144)** |
| 2728pu4F | attgggtaccgggccccccc**gtttaaaC**TTCGGAGTAGCAACGAGTATTTTC |
| 2729pu4R | attgtttagaAGCGCAGTTAATGGTGTATG |
| 2730panF | taactgcgctTCTAAACAATGCCCGCCACC |
| 2731panR | TCCCATACTGCTCCCATAGAAG |
| 2732bapatF | TCTATGGGAGCAGTATGGGATC |
| 2733bapatR | cttcatcaagATGGAACTGATGATCGTCCAG |
| 2734pus1F | tcagttccatCTTGATGAAGGTCTGGGTTG |
| 2735pus1R | tattatcgataccgtcgaccTCCAGACAACCACCCTATCTC |
|  | **Entire new β-alanine 3-HP pathway vector (plasmid vector 3HP4145)** |
| 2736pmbF | atacgaagttattgcagcccGGGACATGCTGGAAGGGATTTTC |
| 2737pmbR | gcacgatcatTTTGAAGATGGATGAGAAGTCGG |
| 2738hpdhF | catcttcaaaATGATCGTGCTGGTCACGGG |
| 2739hpdhR | ctagaactagtggatcccccgtttaaaCCAATGGGATCCCGTAATCAATTG |

**
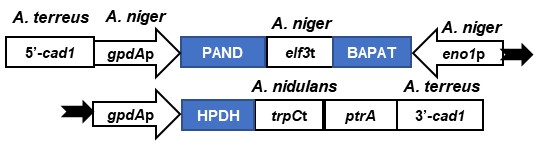

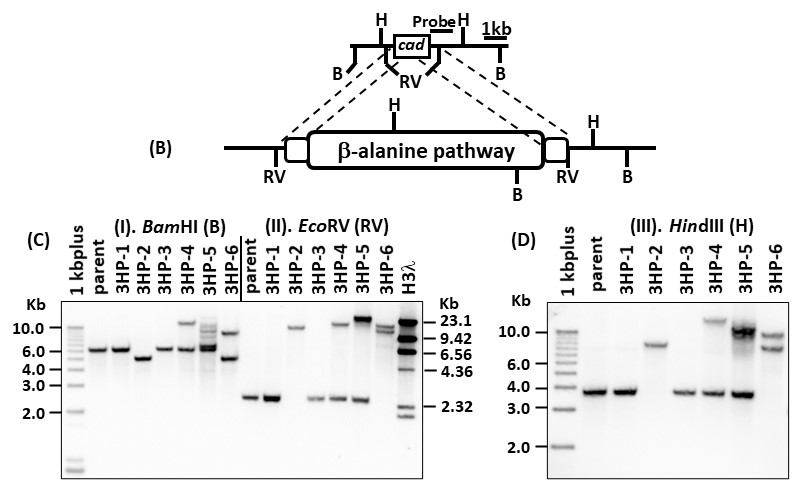
**

**(A)**

**Figure S1. The diagram of the β-alanine 3HP pathway transgene expression cassette with *A. pseudoterreus* *cad1* gene locus targeting and Southern blotting analyses of transgenic *A. pseudoterreus.*** (A) the diagram of the β-alanine 3HP pathway (**3HP4028, Ap3HP)**: 5’-*Cad1*, upstream region of cis-aconitate decarboxylase; *gpdA*p, glyceraldehyde-3-phosphate dehydrogenase promoter from either *A. niger* or *A. nidulans*; PAND, *T. castaneum* aspartate decarboxylase; *elf3*t, *A. niger* elongation factor 3 transcriptional terminator; BAPAT, *B. cereus* β-alanine-pyruvate aminotransferase; *eno1*

p, *A. niger* enolase; HPDH, *E. coli* 3-hydroxypropionate dehydrogenase; *trpC*t, *A. nidulans* trpC transcriptional terminator; *ptrA*, pyrithiamine resistance gene of *A. oryzae*, and 3’-*Cad1*, downstream region of cis-aconitate decarboxylase. (B) the diagram of the β-alanine 3HP pathway with the pattern of restriction endonuclease *Bam*HI, *Eco*RV, and *Hin*dIII. (C) the restriction fragment length polymorphism of *Bam*HI or *Eco*RV in selected transgenic strains. (D) the restriction fragment length polymorphism of *Hin*dIII in selected transgenic strains.

**Figure S2. 3-hydroxypropionic acid and itaconic acid production in the selected individual transgenic strains of *A. pseudoterreus* with over-expression of the β-alanine 3HP pathway transgene expression cassette 3HP4028 (Ap3HP) in the modified RDM medium at 30^o^C and 200 rpm for 7 days.** The strain 2 and 6 were with the homologous recombination of transgene expression cassette Ap3HP at the *cad1* locus (*cad1*Δ), while strain 4 & 5 with the random integration of the transgene cassette into the chromosomes. All data were average of two biological replates.


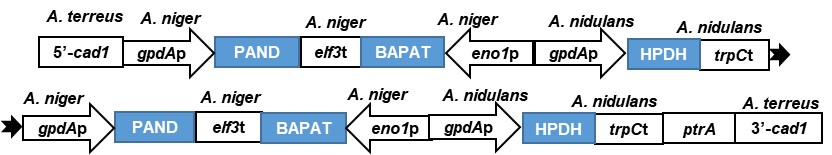


**Figure S3. The diagram of β-alanine 3HP pathway transgene expression cassette with two identical copies of β-alanine 3HP pathway** (**3HP4046, 2x3HP).** 5’-*Cad1*, upstream region of cis-aconitate decarboxylase; *gpdA*p, glyceraldehyde-3-phosphate dehydrogenase promoter from either *A. niger* or *A. nidulans*; PAND, *T. castaneum* aspartate decarboxylase; *elf3*t, *A. niger* elongation factor 3 transcriptional terminator; BAPAT, *B. cereus* β-alanine-pyruvate aminotransferase; *eno1*p, *A. niger* enolase promoter; HPDH, *E. coli* 3-hydroxypropionate dehydrogenase; *trpC*t, *A. nidulans* trpC transcriptional terminator; *ptrA*, pyrithiamine resistance gene of *A. oryzae*, and 3’-*Cad1*, downstream region of cis-aconitate decarboxylase.


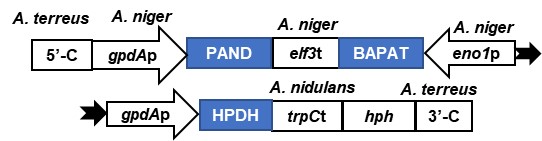


**Figure S4. The diagram of β-alanine 3HP pathway transgene expression cassette with *E. coli* hygromycin B phosphotransferase (*hph*) marker gene** (**3HP4070).** 5’**-**C, 326bp upstream region of cis-aconitate decarboxylase of *A. pseudoterreus*; *gpdA*p, glyceraldehyde-3-phosphate dehydrogenase promoter from either *A. niger* or *A. nidulans*; PAND, *T. castaneum* aspartate decarboxylase; *elf3*t, *A. niger* elongation factor 3 transcriptional terminator; BAPAT, *Bacillus cereus* β-alanine-pyruvate aminotransferase; *eno1*p, *A. niger* enolase promoter; HPDH, *E. coli* 3-hydroxypropionate dehydrogenase; *trpC*t, *A. nidulans* *trpC* transcriptional terminator; *hph*, *E. coli* hygromycin B phosphotransferase gene marker, and 3’**-**C, 283bp upstream region of *ptrA* gene.


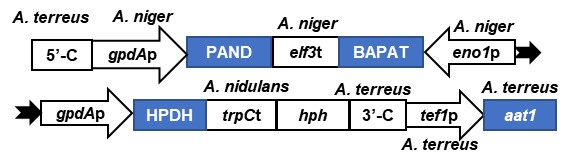


**Figure S5. The diagram of β-alanine 3HP pathway along with an *A. pseudoterreus* *aat1* transgene overexpression under the control of *A. pseudoterreus tef1* gene promoter (3HP4071).** 5’**-**C, 326bp upstream region of cis-aconitate decarboxylase of *A. pseudoterreus*; *gpdA*p, glyceraldehyde-3-phosphate dehydrogenase promoter from either *A. niger* or *A. nidulans*; PAND, *T. castaneum* aspartate decarboxylase; *elf3*t, *A. niger* elongation factor 3 transcriptional terminator; BAPAT, *B. cereus* β-alanine-pyruvate aminotransferase; *eno1*p, *A. niger* enolase promoter; HPDH, *E. coli* 3-hydroxypropionate dehydrogenase; *trpC*t, *A. nidulans* *trpC* transcriptional terminator; *hph*, *E. coli* hygromycin B phosphotransferase gene marker, and 3’**-**C, 283bp upstream region of *ptrA* gene; *tef1*p, translation elongation factor 1 (*tef1*) gene promoter of *A. pseudoterreus*, and *aat1*, *A. pseudoterreus* aspartate aminotransferase gene.


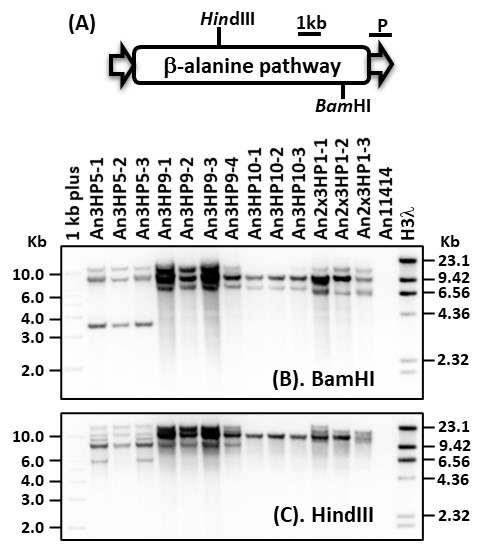


**Figure S6. Southern blot analysis confirmed the β-alanine 3HP pathway random integrations into the chromosomes of *A. nige*r in the selected single spore isolates of transgenic strain An3HP5, An3HP9, An3HP10, and An2x3HP1 with multiple copies of chromosomal insertion.** (A). restriction map of the plasmid DNA fragments containing the β-alanine 3HP pathway used for random integration with restriction endonucleases of ***Bam*HI** (B), or ***Hin*dIII** (C). Southern blot showing the hybridization pattern of the parent strain (no detection) and single spore isolates of transgenic strain An3HP5, An3HP9, An3HP10, and An2x3HP1. The expected sizes of the hybridizing fragments for the restriction endonuclease digestion were >2.34 kb (*Bam*HI) or >7.43 kb (*Hin*dIII). **P** is probe used for Southern blot analysis.


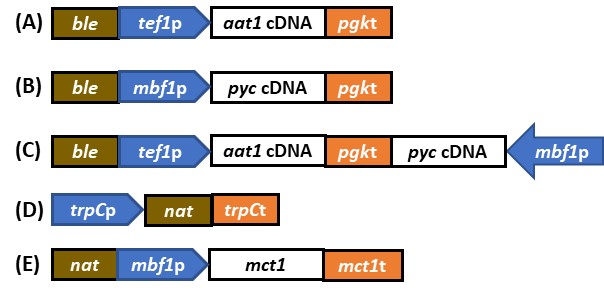


**Figure S7. The diagram of transgene overexpression cassettes of *A. niger* aspartate aminotransferase (*aat1*), pyruvate carboxylase (*pyc*) or their combination, and monocarboxylate transporter (*mct1*).** (A). The *ble*, the bacterial bleomycin resistance gene; *tef1*p, *A. niger* *tef1* gene promoter; *aat1*, *A. niger* aspartate aminotransferase; *pgk*t, *A. niger* *pgk* gene transcriptional terminator (**3HP4074**); (B). & (C) *mbf1*P, *A. niger* *mbf1* gene promoter; *pyc*, *A. niger* *pyc* gene without *aat1* (**3HP4076)** & with *aat1* (**3HP4077**); (D). the *nat1* (*Streptomyces noursei* nourseothricin N-acetyl transferase optimized for the codon usage of *Saccharomyces cerevisiae*) selection marker under the control of *A. nidulans* *trpC* gene promoter and *A. niger* *trpC* gene transcriptional terminator (E). the *nat* selection marker gene; the *mct1*, *A. niger* monocarboxylate transporter; the *mct1*t, *A. niger* *mct1* gene transcriptional terminator.

**Figure S8. 3-hydroxypropionic acid production in the selected individual transgenic strains of *Aspergillus niger* with overexpression of cytosolic aspartate aminotransferase (*aat*, A), pyruvate carboxylase (*pyc*, B), or aspartate aminotransferase + pyruvate carboxylase (*aat*-*pyc*, C) in An3HP9 strain grown in modified RDM medium at 30^o^C and 200 rpm for 7 days.** All data were average of two biological replates.


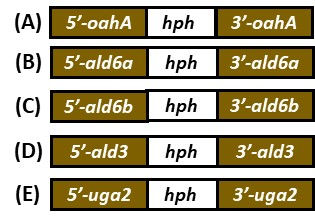


**Figure S9. The diagram of gene disruption constructs of *A. niger* *oahA*, *ald6a*, *ald6b*, *ald3*, and *uga2* gene.** The *hph*, *E. coli* hygromycin B phosphotransferase (*hph*) gene for selection marker; (**A**). *5’-oahA* and *3’-oahA* are DNA fragments of upstream and downstream fragments of *oahA* gene (**3HP4102**); (B) *5’-ald6a* and *3’-ald6a* are DNA fragments of upstream and downstream of *ald6a* gene (**3HP4103**); (C). *5’-ald6b* and *3’-ald6b* are DNA fragments of upstream and downstream of *ald6b* gene (**3HP4104**); (D). *5’-ald3* and *3’-ald3* are DNA fragments of upstream and downstream of *ald3* gene (**3HP4108**); and (E). *5’-uga2* and *3’-uga2* are DNA fragments of upstream and downstream fragments of *uga2* gene (**3HP4109**).


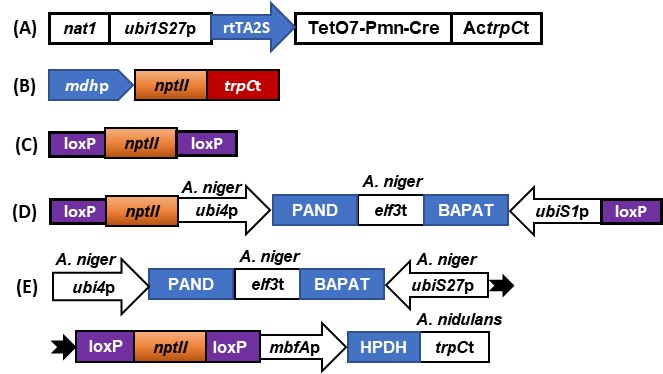


**Figure S10. The diagram of the Tet-On/Cre-loxP (3HP4140) system and the new β-alanine 3HP pathway transgene expression cassette (3HP4145) with loxP-*nptII* marker gene recycle for *A. niger*.** (**A**). the Tet-On/Cre-loxP system (**3HP4140**) for marker gene recycle conditionally activated by doxycycline, *nat1*, *S. noursei* nourseothricin acetyltransferase (resistance) gene; *ubi1S27*p, *A. niger* *ubi1S27* promoter; rtTA2A, the reverse tetracycline transactivator; TetO7, tetracycline resistance operon; Pmn, *A. nidulans* *gpdA* minimal promoter; Cre, Cre recombinase; Ac*trpC*t, *A. carbonarius* *trpC* transcriptional terminator; (**B**). the bacterial neomycin-resistance (*nptII*) marker gene under the control of *A. niger* malate dehydrogenase (*mdh*p) promoter and *A. nidulans* *trpC* transcriptional terminator (*trpC*t) (**3HP4134**); (**C**). the *nptII* marker gene cassette with 31 bp loxP fragments fused at 5’- and 3’-end of its marker gene cassette (**3HP3136**); (**D**). the intermediate transgene expression cassette (**3HP4144**) contains the *ubi4*p, *A. niger* *ubi4* gene promoter; PAND, *T. castaneum* aspartate decarboxylase; *elf3*t, *A. niger* elongation factor 3 transcriptional terminator; BAPAT, *B. cereus* β-alanine-pyruvate aminotransferase; and *ubiS*p, *A. niger* *ubi1S* promoter. (**E**). the final new β-alanine 3HP pathway (**3HP4145**) was assembled with the HPDH, *E. coli* 3-hydroxypropionate dehydrogenase under the control of *A. niger* *mbfA* promoter (*mbfA*p) and *A. nidulans* *trpC* transcriptional terminator(*trpC*t).


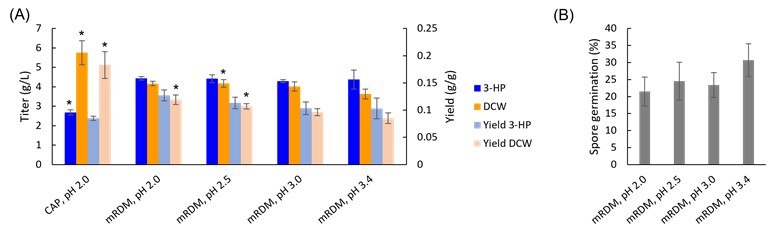


**Figure S11.** **The effects of culture medium and pH on 3-HP production and spore germination in *A. niger* strain An3HP9 grown at 30^o^C and 200 rpm for 7 days.** (A) 3-HP and DCW titer and yield and (B) the percentage of spore germination. The data is the average of three biological replicates. Asterisks indicate statistically significant differences (*p* < 0.05) from mRDM, pH 3.4.
